# Supplementary material for: Variant PRC1 Complex-Dependent H2A Ubiquitylation Drives PRC2 Recruitment and Polycomb Domain Formation
Source: Cell. 2014 Jun 5;157(6):1445–59. doi: 10.1016/j.cell.2014.05.004 (PMC4048464; doi:10.1016/j.cell.2014.05.004)
Supplement: Document S1. Article plus Supplemental Information [file mmc1.pdf]

Cell

Variant PRC1 Complex-Dependent H2A Ubiquitylation Drives PRC2 Recruitment and Polycomb Domain Formation

Neil P. Blackledge,[1,5] Anca M. Farcas,[1,5] Takashi Kondo,[2,5] Hamish W. King,[1] Joanna F. McGouran,[3] Lars L.P. Hanssen,[1] Shinsuke Ito,[2] Sarah Cooper,[4] Kaori Kondo,[2] Yoko Koseki,[2] Tomoyuki Ishikura,[2] Hannah K. Long,[1] Thomas W. Sheahan,[1] Neil Brockdorff,[4] Benedikt M. Kessler,[3] Haruhiko Koseki,[2] and Robert J. Klose[1,*]
[1]Laboratory of Chromatin Biology and Transcription, Department of Biochemistry, University of Oxford, Oxford, OX1 3QU, UK
[2]Laboratory of Developmental Genetics, RIKEN Center for Integrative Medical Sciences, Yokohama, Kanagawa 230-0045, Japan
[3]Ubiquitin Proteolysis Group, Central Proteomics Facility, Target Discovery Institute, Nuffield Department of Medicine, University of Oxford, OX3 7BN, UK
[4]Laboratory of Developmental Epigenetics, Department of Biochemistry, University of Oxford, Oxford, OX1 3QU, UK
[5]Co-first author
*Correspondence: rob.klose@bioch.ox.ac.uk
http://dx.doi.org/10.1016/j.cell.2014.05.004
This is an open access article under the CC BY license (http://creativecommons.org/licenses/by/3.0/).

SUMMARY

Chromatin modifying activities inherent to polycomb repressive complexes PRC1 and PRC2 play an essential role in gene regulation, cellular differentiation, and development. However, the mechanisms by which these complexes recognize their target sites and function together to form repressive chromatin domains remain poorly understood. Recruitment of PRC1 to target sites has been proposed to occur through a hierarchical process, dependent on prior nucleation of PRC2 and placement of H3K27me3. Here, using a de novo targeting assay in mouse embryonic stem cells we unexpectedly discover that PRC1-dependent H2AK119ub1 leads to recruitment of PRC2 and H3K27me3 to effectively initiate a polycomb domain. This activity is restricted to variant PRC1 complexes, and genetic ablation experiments reveal that targeting of the variant PCGF1/PRC1 complex by KDM2B to CpG islands is required for normal polycomb domain formation and mouse development. These observations provide a surprising PRC1-dependent logic for PRC2 occupancy at target sites in vivo.

INTRODUCTION

In eukaryotic cells, chromatin structure and posttranslational modification of histone proteins play central roles in regulating gene expression. This is exemplified in animals where polycomb group proteins function as chromatin-based transcriptional repressors through their capacity to catalyze histone modifications and form higher order chromatin structures (recently reviewed in Schwartz and Pirrotta, 2013; Simon and Kingston, 2013). Loss of polycomb protein function in *Drosophila* leads to abnormal body plan specification and in vertebrates polycomb orthologs are essential for normal embryonic development. Polycomb proteins are also perturbed in a range of cancers, suggesting that the polycomb system is critical for maintenance of normal cell identity (Bracken and Helin, 2009).

Polycomb proteins are generally found in one of two protein complexes, the polycomb repressive complexes 1 or 2 (PRC1 or PRC2). In mammals, the catalytic core of PRC2 is comprised of EZH1 or EZH2, which trimethylate histone H3 on lysine 27 (H3K27me3) (Cao et al., 2002; Czermin et al., 2002; Kuzmichev et al., 2002; Müller et al., 2002). A series of auxiliary proteins, including SUZ12 and EED, associate with EZH1/2 and modulate targeting, chromatin binding, and catalytic activity (Cao and Zhang, 2004; Ketel et al., 2005; Margueron et al., 2009; Pasini et al., 2004). In contrast, PRC1 monoubiquitylates histone H2A on lysine 119 (H2AK119ub1) (de Napoles et al., 2004; Wang et al., 2004a). The catalytic core of PRC1 consists of RING1A or RING1B, which dimerize with one of six PCGF protein partners (PCGF1-6) that regulate assembly of specific PRC1 complexes (Buchwald et al., 2006; Chamberlain et al., 2008; Farcas et al., 2012; Gao et al., 2012; Gearhart et al., 2006; Li et al., 2006; Ogawa et al., 2002; Sánchez et al., 2007). Together, the combined activities of PRC1 and PRC2 are thought to be essential for normal polycomb-mediated transcriptional repression and developmental gene regulation (recently reviewed in Simon and Kingston, 2013). Nevertheless, the molecular mechanisms by which polycomb group proteins recognize their target sites and initiate repressive chromatin domains remain poorly defined.

Molecular and functional characterization of the polycomb repressive complexes has revealed that they do not function independently (Bracken et al., 2006; Ku et al., 2008; Papp and Müller, 2006; Schwartz et al., 2006). Instead, H3K27me3 placed by PRC2 is recognized by PRC1 complexes that contain chromobox (CBX) proteins (Cao et al., 2002; Min et al., 2003; Wang

et al., 2004b). Based on these initial observations, the prevailing view over the past decade has been that PRC1 is recruited in a hierarchical manner to sites with pre-existing PRC2 activity and H3K27me3. However, it has recently emerged that CBX proteins are in direct competition with two additional factors, RYBP/YAF2, for a mutually exclusive binding site on RING1A/B (Wang et al., 2010). Significantly, H3K27me3-binding CBX proteins are limited to canonical PRC1 complexes containing either PCGF2 (MEL18) or PCGF4 (BMI1) and the Polyhomeotic proteins (PHC1/2/3) (Gao et al., 2012; Levine et al., 2002), while all PCGF proteins interact with RYBP/YAF2 to form variant PRC1 complexes lacking CBX proteins (Farcas et al., 2012; Gao et al., 2012; Gearhart et al., 2006; Lagarou et al., 2008; Sánchez et al., 2007; Tavares et al., 2012) (Figure 1A). The identification of variant PRC1 complexes and the observation that RING1B can occupy many of its target sites in the absence of H3K27me3 suggests that the hierarchical recruitment mechanism cannot explain all PRC1 complex targeting (Schoeftner et al., 2006; Tavares et al., 2012). Therefore, the central principles that underpin recognition of polycomb target sites in vivo and the molecular chain of events that leads to the formation of polycomb domains integrating both PRC1 and PRC2 activity remain unclear.

In this study, we utilize a de novo targeting system and discover, contrary to expectation based on the proposed hierarchical recruitment mechanism, that binding of variant PRC1 complexes to chromatin is sufficient to initiate the formation of a polycomb domain containing PRC2 and H3K27me3. This activity is inherent to variant PRC1 complexes and relies on H2AK119ub1. Building on this striking observation, genetic ablation approaches in mouse embryonic stem cells (ESCs) reveal that deletion of PRC1 has dramatic genome-wide effects on PRC2 occupancy and H3K27me3. We further demonstrate that recognition of nonmethylated DNA by KDM2B, part of the PCGF1/PRC1 variant complex, is important for deposition of H2AK119ub1 and recruitment of PRC2 to a subset of CpG island targets and that this targeting activity is essential for normal mouse development. Together, these observations reveal a new PRC1-dependent logic for polycomb domain formation.

RESULTS

A System to Target PRC1 to Chromatin De Novo

The hierarchical recruitment model posits that PRC2-dependent H3K27me3 is required to recruit canonical PRC1 complexes to chromatin. The recent demonstration that variant PRC1 complexes bind to many target sites, albeit at lower levels, independently of H3K27me3 suggests that PRC1 function may be more complex than previously envisaged (Tavares et al., 2012). This new insight necessitates a more detailed examination of mammalian PRC1 complex function and targeting in vivo. Therefore, we sought to design a system in which individual PRC1 complexes could be targeted de novo to a chromatin environment free from the complexities and regulatory influences of natural polycomb target sites. To achieve this, a large bacterial artificial chromosome (BAC) containing human DNA that lacks identifiable genes and shows no evidence for gene-, enhancer-, or polycomb-associated chromatin modifications was selected and bacterial Tet operator (TetO) DNA-binding sites were centrally inserted (Figure 1B). Importantly, the TetO lacks CpG dinucleotides and has no resemblance to natural polycomb targets which are CpG-rich (Ku et al., 2008). The TetO BAC was transposed into mouse ES cells at a site on chromosome 8, effectively flanking the TetO array with long stretches of inert chromatin (Figures 1B, S1A, and S1B). Fusion of PRC1 components to the bacterial Tet repressor (TetR) DNA-binding domain would permit de novo recruitment to the TetO array (Figure 1C) and the direct consequences of fusion protein occupancy could be examined by chromatin immunoprecipitation (ChIP).

Variant PRC1 Complexes Place H2AK119ub1 and Recruit PRC2

In mammals, PCGF proteins (PCGF1-6) are thought to define the composition of individual PRC1 complexes and regulate their assembly and function (Figure 1A). To dissect how individual PRC1 complexes function on chromatin, PCGF1-5 were fused to TetR and stably expressed in the TetO cell line (Figure 1C). ChIP experiments revealed that TetR-PCGF fusion proteins bound the TetO array and diminished to background levels in flanking regions (Figure 1D). All PCGF proteins resulted in recruitment of RING1B, but surprisingly, only PCGF1, 3, and 5, which exclusively form variant PRC1 complexes (Farcas et al., 2012; Gao et al., 2012; Gearhart et al., 2006; Sánchez et al., 2007), placed significant levels of H2AK119ub1 (Figure 1D).

PRC1 and PRC2 largely occupy a common set of target sites in vivo (Ku et al., 2008; Papp and Müller, 2006; Schwartz et al., 2006; Tolhuis et al., 2006), and this has been attributed to the hierarchical recruitment mechanism. Therefore, the possibility that PRC1 could potentially drive a reciprocal process and mediate PRC2 occupancy has not been specifically examined. By directly targeting individual PRC1 complexes to the TetO array, a unique opportunity existed to test whether PRC1 complexes can also drive PRC2 occupancy. Surprisingly, in the TetR-PCGF1, 3, and 5 fusion lines, ChIP analysis revealed binding of PRC2 components and H3K27me3 in regions flanking the TetO (Figure 1E). In contrast, TetR fusions with PCGF proteins that can form canonical PRC1 complexes (PCGF2 and 4) resulted in little, if any, PRC2 targeting and H3K27me3 (Figure 1E). Strikingly, variant complex-dependent PRC2 recruitment and H3K27me3 was also observed at a single naturally occurring TetO site in the mouse genome (Figures S1C and S1D), indicating that this is not unique to the engineered TetO array. Therefore, contrary to expectation based on the hierarchical recruitment mechanism, de novo recruitment of the PCGF 1, 3, and 5 variant PRC1 complexes results in the formation of a polycomb domain containing PRC2 and H3K27me3.

The Hierarchical PRC2-Dependent Recruitment of Canonical PRC1 Complexes Fails to Place H2AK119ub1

Surprisingly, PCGF proteins that form canonical PRC1 complexes appeared less competent at H2AK119ub1 placement in tethering assays (Figure 1D). This lack of activity could be inherent to canonical PRC1 complexes or possibly result from their covalent fusion to TetR. To circumvent the necessity to fuse canonical complexes to TetR, PRC2 was recruited to the TetO via a TetR-EED fusion (Figure 2A) (Hansen et al., 2008). This led to deposition of H3K27me3 and recruitment of

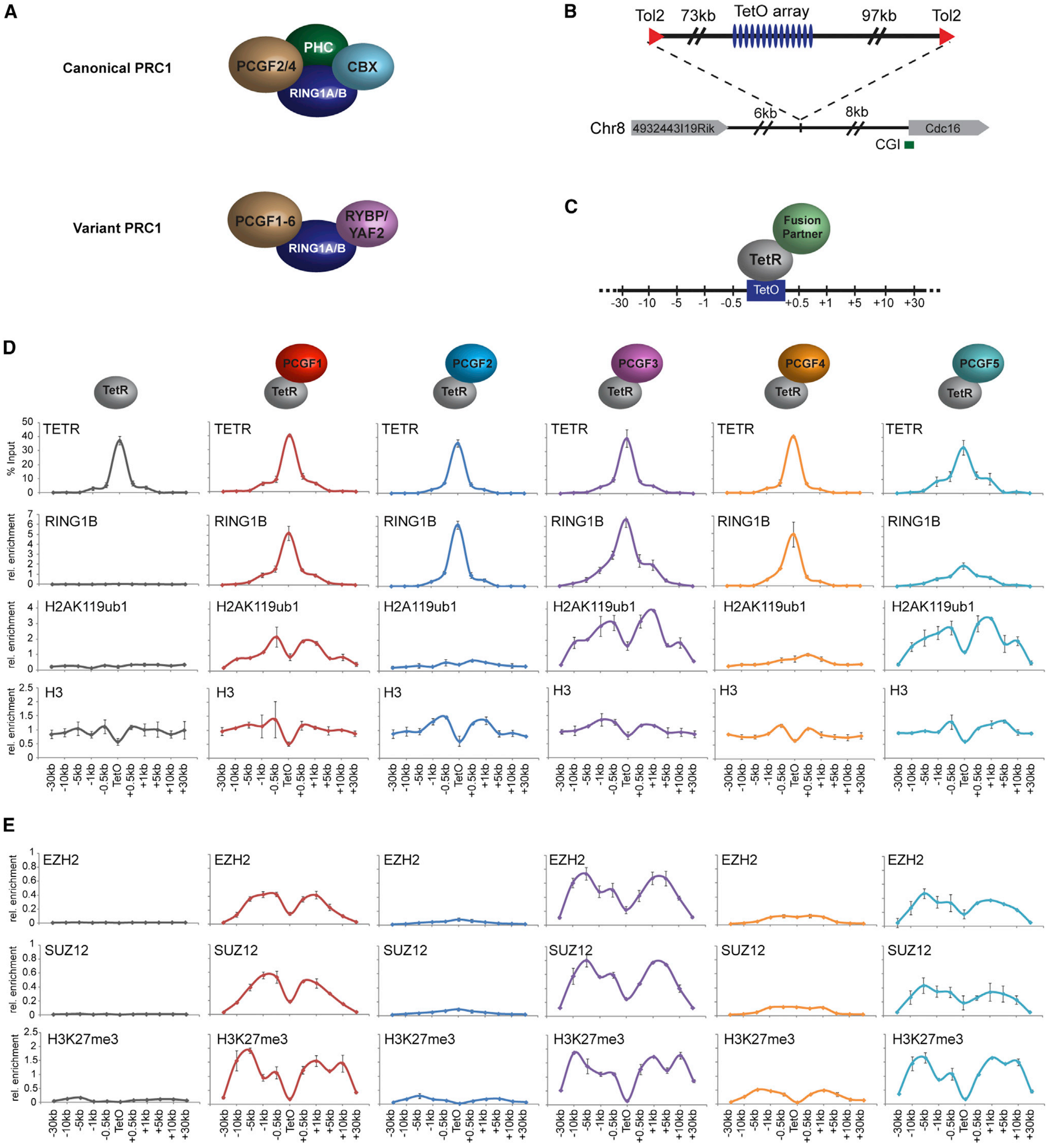

Figure 1. PCGF 1, 3, and 5 Variant PRC1 Complexes Catalyze H2AK119ub1 and Create A Polycomb Domain Containing PRC2 and H3K27me3

(A) A schematic illustrating the core components of canonical and variant PRC1 complexes.

(B) The TetO array at its integration site on mouse chromosome 8.

(C) Targeting of factors to the TetO via the TetR DNA-binding domain. Numbers represent qPCR primer positions (kb) with respect to TetO array.

(D) ChIP analysis for fusion protein occupancy (TetR), RING1B, H2AK119ub1, and histone H3 across the TetO containing locus. Fusion protein identity is indicated above each panel.

(E) As in (D) ChIP analysis for PRC2 components and H3K27me3. All ChIP experiments were performed at least in biological duplicate with error bars showing standard error of the mean (SEM).

See also Figure S1.

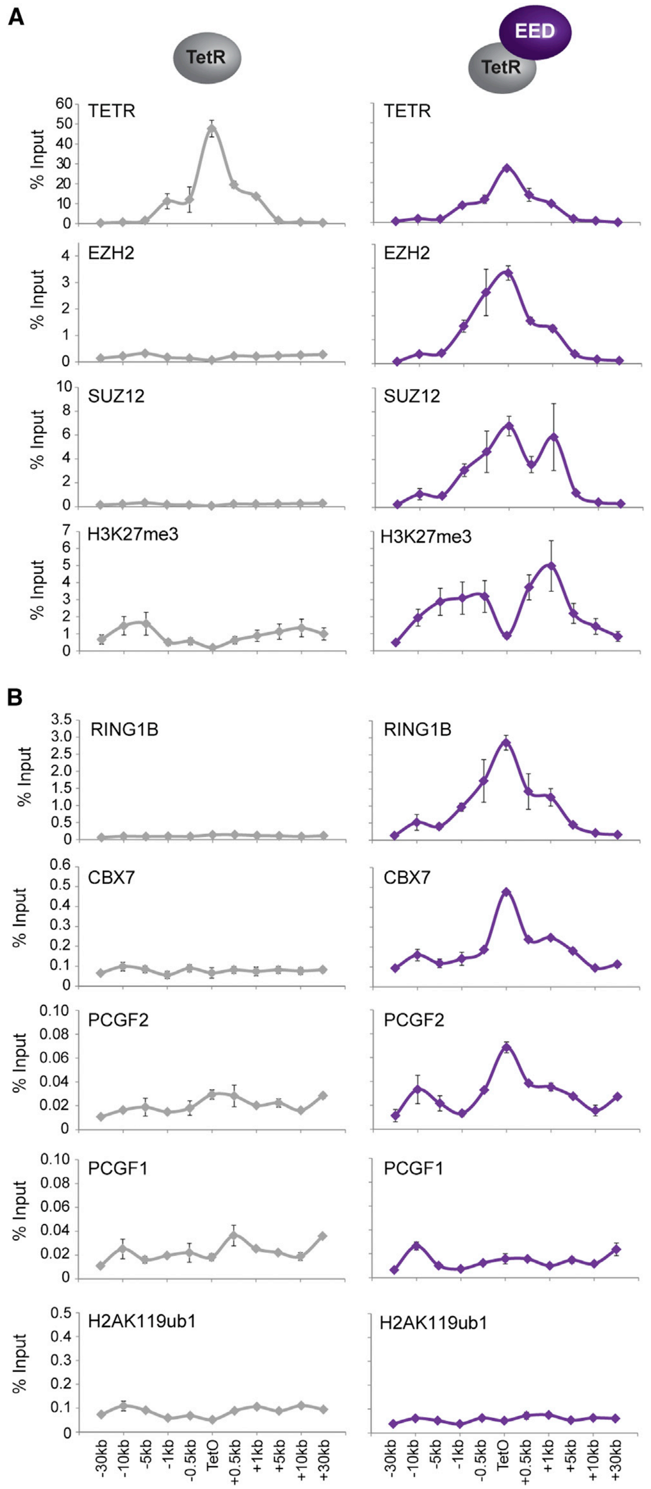

Figure 2. Hierarchical Recruitment of Canonical PRC1 Fails to Result in H2AK119ub1

(A) ChIP analysis for fusion protein occupancy (TetR), PRC2 components, and H3K27me3 across the TetO-containing locus in lines expressing TetR alone and a TetR-EED fusion.

(B) ChIP analysis for PRC1 components and H2AK119ub1 performed as described in (A).

All ChIP experiments in (A) and (B) were performed at least in biological duplicate with error bars showing SEM.

endogenous PCGF2 and CBX7, but not PCGF1, suggesting PRC2-dependent recruitment of canonical PRC1 complexes (Figure 2B). As was the case with direct tethering of PCGF2 or 4, native canonical PRC1 complex nucleation failed to deposit H2AK119ub1, suggesting the lack of activity in canonical PRC1 tethering experiments does not result from TetR fusion (Figure 2B). Interestingly, the binding profiles for canonical PRC1 components were not completely coincident with H3K27me3, as might be expected if occupancy was entirely CBX dependent. It remains unclear why this disparity in profiles existed, but it may result from secondary structural effects driven by exclusive canonical PRC1 complex recruitment (Isono et al., 2013) or other undefined mechanisms involved in canonical PRC1 recruitment to regions containing PRC2 and H3K27me3. Interestingly, a similar discordance between CBX (PC) protein binding and H3K27me3 was observed at polycomb target sites in *Drosophila* cell culture models (Schwartz et al., 2006). Nevertheless, this apparent failure of PRC2 and H3K27me3 to direct H2AK119ub1 parallels observations in mouse ESC lines devoid of H3K27me3 where levels of H2AK119ub1 at polycomb target sites are largely unaffected (Schoeftner et al., 2006; Tavares et al., 2012). Together, these observations strongly suggest that PRC2-mediated recruitment of canonical PRC1 complexes fails to catalyze significant levels of H2AK119ub1.

Deposition of H2AK119ub1 Is Sufficient to Nucleate PRC2 and H3K27me3

The unexpected observation that variant PRC1 complexes can nucleate PRC2 to establish a polycomb domain de novo suggests that a feature associated with variant complex occupancy, perhaps H2AK119ub1, is responsible for this activity (Figure 1). To test this possibility, a single polypeptide fusion between the dimerization domains of RING1B and PCGF4 was engineered and fused to TetR (Bentley et al., 2011; Buchwald et al., 2006; Li et al., 2006). This minimal RING1B/PCGF4 catalytic domain (RPCD) does not form normal PRC1 complexes (Figure 3A) but retains H2AK119ub1 E3 ligase activity (Figure 3B) (Cooper et al., 2014) leading to a striking enrichment of EZH2, SUZ12, and H3K27me3 at the TetO (Figure 3B). When mutations were engineered in TetR-RPCD (TetR-RPCDmut), rendering it incapable of catalyzing H2AK119ub1, PRC2 and H3K27me3 were no longer recruited to the TetO (Figure 3B). This suggests that H2AK119ub1, and not simply binding of PRC1 complexes, is the central determinant driving PRC1-dependent recruitment of PRC2.

Deletion of PRC1 and Loss of H2AK119ub1 Affects PRC2 Occupancy and H3K27me3 Genome-wide

To examine the possibility that H2AK119ub1 may play a general role in PRC2 localization and activity at normal polycomb target sites, we exploited a *Ring1a*$^{-/-}$*Ring1b*$^{fl/fl}$ mouse ESC system, in which H2AK119ub1 can be rapidly depleted by removing the catalytic core of all PRC1 complexes (RING1A/B) through addition of the drug tamoxifen, without disrupting the cellular protein

A

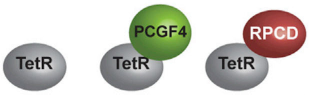

	TETR IP		TETR-PCGF4 IP		TETR-RPCD IP	
Name	Score	Cov (%)	Score	Cov (%)	Score	Cov (%)
TETR	391	55	380	52	704	55
RING1B	–	–	4765	42	8162	38
PCGF4	–	–	1033	41	1091	14
RYBP			1189	48	351	19
RING1A			1546	38		
PHC1			1148	34		
CBX2			443	25		
CBX6			135	8		
CBX7			51	17		
YAF2			29	19		

B

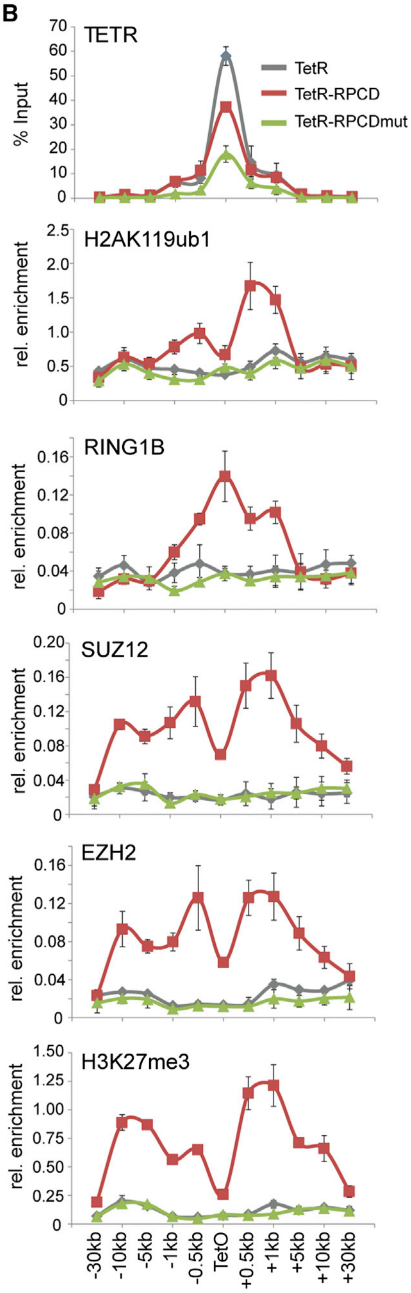

Figure 3. H2AK119ub1 Is Required for PRC1-Dependent Recruitment of PRC2 and H3K27me3

(A) Mass spectrometry analysis of purified TetR-PCGF4 and TetR-RPCD (minimal RING1B/PCGF4 catalytic domain) proteins. The mascot score and percentage coverage is indicated for polycomb group proteins in each sample.

(B) ChIP analysis for PRC1, PRC2 and their respective modifications in cell lines expressing TetR, TetR-RPCD, and TetR-RPCDmut. All ChIP experiments were performed at least in biological duplicate with error bars showing SEM.

levels of PRC2 components (Endoh et al., 2008) (Figures 4A–4C). Following RING1A/B deletion, ChIP-sequencing revealed a clear loss of SUZ12, EZH2, and H3K27me3 at individual genes (Figures 4D and S2A) and at target sites genome-wide (Figure 4E and 4F). Indeed, 85% of SUZ12 and 83% of EZH2 sites showed a greater than 1.5-fold reduction in occupancy after PRC1 removal (Figures 4G and S3A). A closer inspection of SUZ12 sites defined as having a less than 1.5-fold change in PRC2, revealed that these sites do exhibit an observable loss in PRC2 binding (Figure S3B, S3C, and S3D) suggesting that most PRC2 sites are affected by loss of PRC1 activity. These effects on PRC2 occupancy were seemingly independent of high-level gene reactivation, as PRC2 reductions occurred at genes displaying small or large fluctuations in gene expression (Figures S2B and S2C).

Previous studies report that specific polycomb target sites rely on transcription factors or long noncoding RNAs (lncRNAs) for normal polycomb protein recruitment (Simon and Kingston, 2013). In mouse ESCs the transcription factor REST (Arnold et al., 2013; Dietrich et al., 2012) and the *Meg3* lncRNA (Kaneko et al., 2014) are thought to contribute to these targeting events. Interestingly, following PRC1 deletion, PRC2 occupancy was reduced at REST-occupied PRC2 sites (Figure S3E) and *Meg3* lncRNA targets (Figure S3F), suggesting these mechanisms are insufficient to maintain normal levels of PRC2 and H3K27me3 in the absence of PRC1. Further segregation of PRC2 sites into those existing in a "bivalent" state containing H3K4me3 and H3K27me3 revealed that this subset of PRC2 sites had a slightly larger fold change in PRC2 occupancy following PRC1 deletion (Figure S3G). However, this difference was modest compared to the overall magnitude of PRC2 loss observed at both bivalent and nonbivalent sites.

Unfortunately, it was not possible to examine whether long-term ablation of PRC1 activity would lead to a complete loss of PRC2 occupancy on chromatin, because mouse ESCs completely lacking PRC1 (Endoh et al., 2008), unlike those lacking PRC2 (Boyer et al., 2006; Chamberlain et al., 2008; Leeb et al., 2010; Pasqualucci et al., 2011), cannot be continuously maintained in culture. Under the conditional deletion conditions used here, some degree of residual PRC1 is evident (Figure 4D, 4E, and S2A), and this may contribute to the remaining PRC2 occupancy. It should be noted, however, that PRC1-independent PRC2 targeting activities could also contribute to this residual PRC2 occupancy (Simon and Kingston, 2013) (see Discussion). Nevertheless, to examine in more detail the relationship between PRC1 loss and the resulting reduction in PRC2 occupancy, the fold change in PRC1 and PRC2 was compared at individual target sites genome-wide. This revealed a striking genome-wide correlation between the magnitude of PRC1 and PRC2 loss (Figures 4H and S3H), suggesting that

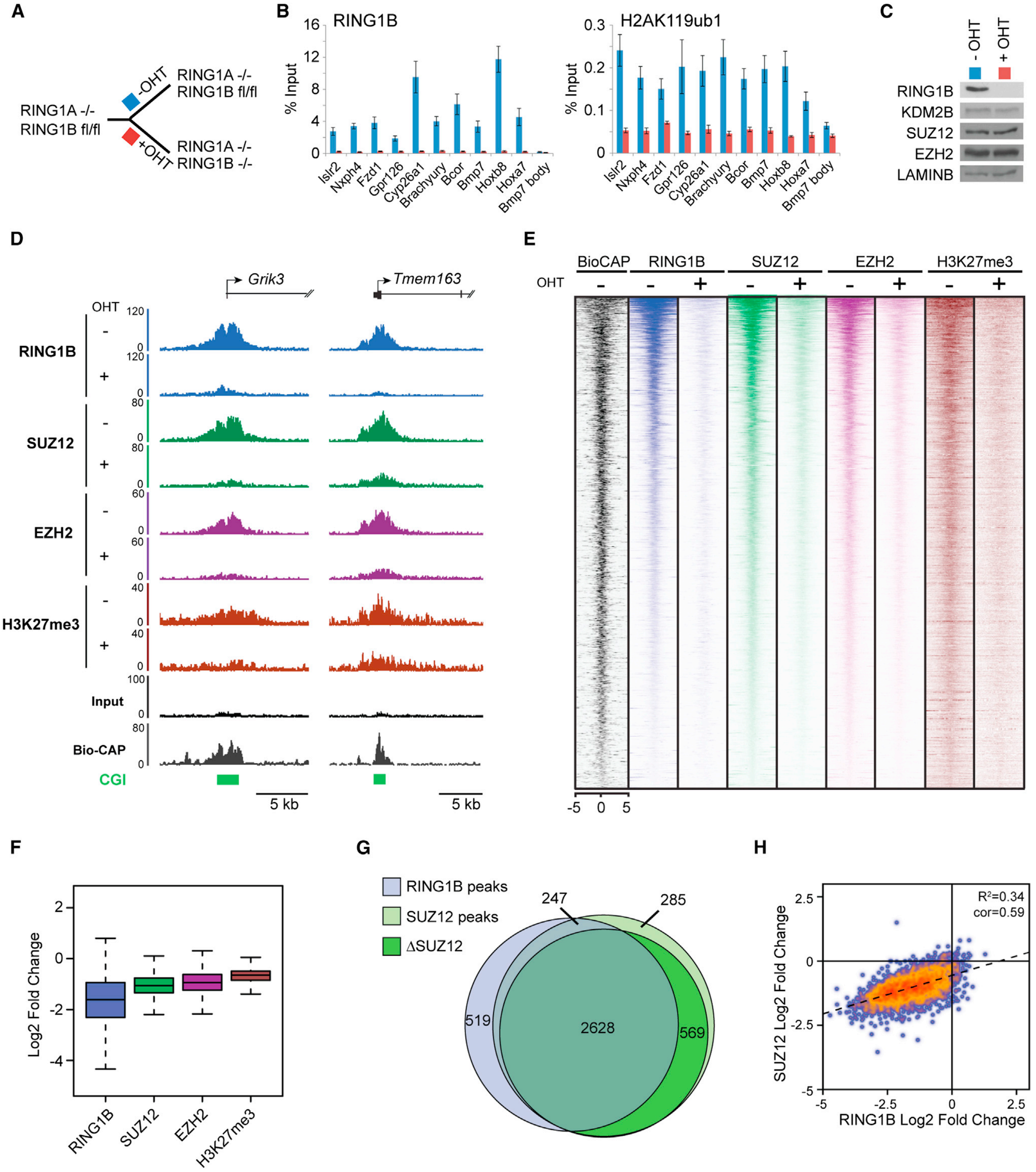

Figure 4. PRC1 Has a Genome-wide Role in PRC2 Recruitment and Polycomb Domain Formation at Target Sites in Mouse ESCs

(A) A schematic of the *Ring1a*$^{-/-}$*Ring1b*$^{fl/fl}$ system in which addition of tamoxifen (OHT) leads to deletion of RING1A/B.

(B) ChIP analysis for RING1B and H2AK119ub1 at polycomb target sites before (−OHT) and after 48 hr (+OHT) tamoxifen treatment. ChIP experiments were performed at least in biological duplicate with error bars showing SEM.

(C) Western blot analysis of polycomb factors following deletion of RING1A/B.

(legend continued on next page)

PRC1 and H2AK119ub1 are central players in normal PRC2 nucleation.

KDM2B Recruits the Variant PCGF1/PRC1 Complex to Create a PRC2-Containing Polycomb Domain

Deletion of RING1A/B in mouse ESCs supports a model whereby H2AK119ub1 contributes to the occupancy of PRC2 at natural target sites in vivo. However, removal of RING1A/B disrupts both canonical and variant PRC1 complex activity. Understanding if variant PRC1 complexes can drive this process at natural target sites is challenging, as variant PRC1 complex targeting mechanisms remain poorly defined. An exception is the PCGF1/PRC1 complex which contains a histone lysine demethylase protein, KDM2B, which binds to nonmethylated DNA via a ZF-CxxC DNA-binding domain (Farcas et al., 2012; He et al., 2013; Long et al., 2013; Wu et al., 2013). Nonmethylated DNA is generally concentrated in vertebrate regulatory elements called CpG islands, and most mammalian polycomb target sites are associated with CpG islands (Ku et al., 2008). KDM2B may therefore represent a direct molecular link between recognition of CpG island target sites and occupancy of both PRC1 and PRC2. To determine if KDM2B binding is sufficient to recruit the PCGF1/PRC1 complex and establish a polycomb domain de novo, a TetR-KDM2B fusion protein was stably expressed in the TetO cell line (Figure 5A). TetR-KDM2B led to RING1B, PCGF1, and H2AK119ub1 deposition (Figure 5A). This was not observed with the related KDM2A protein which does not interact with PRC1 (Figure 5A) (Blackledge et al., 2010). Importantly, PCGF1/PRC1 recruitment by KDM2B resulted in binding of PRC2 and H3K27me3 (Figures 5A and S4C). This activity was dependent on recruitment of PCGF1/PRC1, as depletion of PCGF1 in the TetR-KDM2B line caused a clear reduction in both PRC1 and PRC2 (Figures 5B and 5C). Interestingly, polycomb domain formation did not rely on KDM2B demethylase activity, as a catalytic mutant of KDM2B or a short form of the protein that lacks the demethylase domain recruited PRC1 and PRC2 to similar levels (Figures S4A–S4C). Therefore, de novo targeting of the PCGF1/PRC1 complex by KDM2B leads to polycomb domain formation in a manner similar to TetR-PCGF1 (Figure 1).

A System to Inducibly Ablate Targeting of the Variant PCGF1/PRC1 Complex by KDM2B

The observation that KDM2B can nucleate PRC1 and PRC2 provided an opportunity to examine whether targeting of the PCGF1/PRC1 variant complex to natural CpG island target sites is important for polycomb domain formation. To achieve this, a novel genetic system was designed in which an exon that encodes most of the KDM2B ZF-CxxC domain and is shared by both the long and short form of the protein (Figures S4 and S5A) (Fukuda et al., 2011) was flanked by loxP sites (*Kdm2b*$^{fl/fl}$) (Figure 5D). Homozygous *Kdm2b*$^{fl/fl}$ mouse ESC lines were then derived that also stably express a tamoxifen-inducible form of Cre-recombinase. Addition of tamoxifen rapidly yielded KDM2B long and short form proteins that lack the ZF-CxxC domain (Figures 5E and S5B), but remain associated with the PCGF1/PRC1 variant complex (Figure S5D). Importantly, cellular levels of PRC1 and PRC2 components were unaffected (Figure S5C). ChIP-seq for KDM2B in the *Kdm2b*$^{fl/fl}$ cells revealed KDM2B occupancy at CpG islands as previously described (Figure 5F) (Farcas et al., 2012; He et al., 2013; Wu et al., 2013). However, tamoxifen-mediated deletion of the ZF-CxxC domain caused a near complete loss of KDM2B chromatin occupancy and removal of PCGF1 from CpG islands (Figures 5F and 5G). Therefore, deletion of the KDM2B ZF-CxxC domain is sufficient to ablate normal targeting of the PCGF1/PRC1 complex in vivo.

KDM2B-Mediated PCGF1/PRC1 Targeting Is Required for Normal Recruitment of PRC2 and Polycomb Domain Formation at a Subset of CpG Island Sites

To identify polycomb sites that are dependent on KDM2B-mediated targeting for RING1B binding, RING1B ChIP-seq was carried out in the *Kdm2b*$^{fl/fl}$ and tamoxifen treated cells. Removal of the KDM2B ZF-CxxC domain resulted in a widespread reduction of RING1B chromatin binding (Figures 6A–6D). Of the 3,488 high-confidence RING1B peaks identified in ESCs, 43% showed a greater than 1.5-fold reduction in RING1B occupancy after tamoxifen treatment (Figure 6D), suggesting that a subset of RING1B-bound CpG islands is most sensitive to KDM2B loss, and other PRC1 complexes must contribute to RING1B occupancy at the remaining sites. Importantly, sites exhibiting RING1B loss also showed reduced H2AK119ub1 levels, consistent with a role for PCGF1/PRC1 in catalyzing this modification (Figure 6I).

When SUZ12 ChIP-seq was carried out in the *Kdm2b*$^{fl/fl}$ and tamoxifen treated cells, there was a striking reduction of SUZ12 occupancy which broadly corresponded to the reduction in RING1B (Figures 6A–6D). Indeed, 84% of SUZ12 peaks showing a greater than 1.5-fold reduction in SUZ12 binding overlapped with RING1B peaks (Figure 6D), and 78% of these regions were associated with a greater than 1.5-fold reduction in RING1B binding. This again suggests an intimate relationship between loss of KDM2B-mediated PCGF1/PRC1 targeting and PRC2 occupancy. To examine this more closely, the relative change in the levels of RING1B and SUZ12 were directly compared (Figures 6E and 6F). Strikingly, as with the

(D) Snapshots of ChIP-seq traces for RING1B, SUZ12, EZH2, and H3K27me3 in the *Ring1a*$^{-/-}$*Ring1b*$^{fl/fl}$ cells prior to (−OHT) and following 48 hr +OHT treatment at the *Grik3* and *Tmem163* genes. Bio-CAP indicates nonmethylated DNA and CpG islands (CGI) are shown as green bars.

(E) Heat map analysis of SUZ12 peaks (n = 3,819), showing ChIP-seq data for RING1B, SUZ12, EZH2, and H3K27me3 at a 10 kb region centered over the SUZ12 peaks prior to (−OHT) and after 48 hr +OHT treatment. Bio-CAP is included to indicate nonmethylated DNA at these sites.

(F) A box and whisker plot showing log2 fold changes in normalized read counts comparing the ChIP-seq signal at SUZ12 sites −OHT and after 48 hr +OHT treatment.

(G) A Venn diagram showing the overlap of RING1B (light blue) and SUZ12 (light green) peaks including a further segregation of SUZ12-bound regions that show a greater than 1.5-fold change in SUZ12 occupancy (ΔSUZ12, dark green) after 48 hr tamoxifen treatment (+OHT).

(H) A scatter plot comparing the fold change of RING1B and SUZ12 at SUZ12 peaks −OHT and after 48 hr +OHT treatment.

See also Figures S2 and S3.

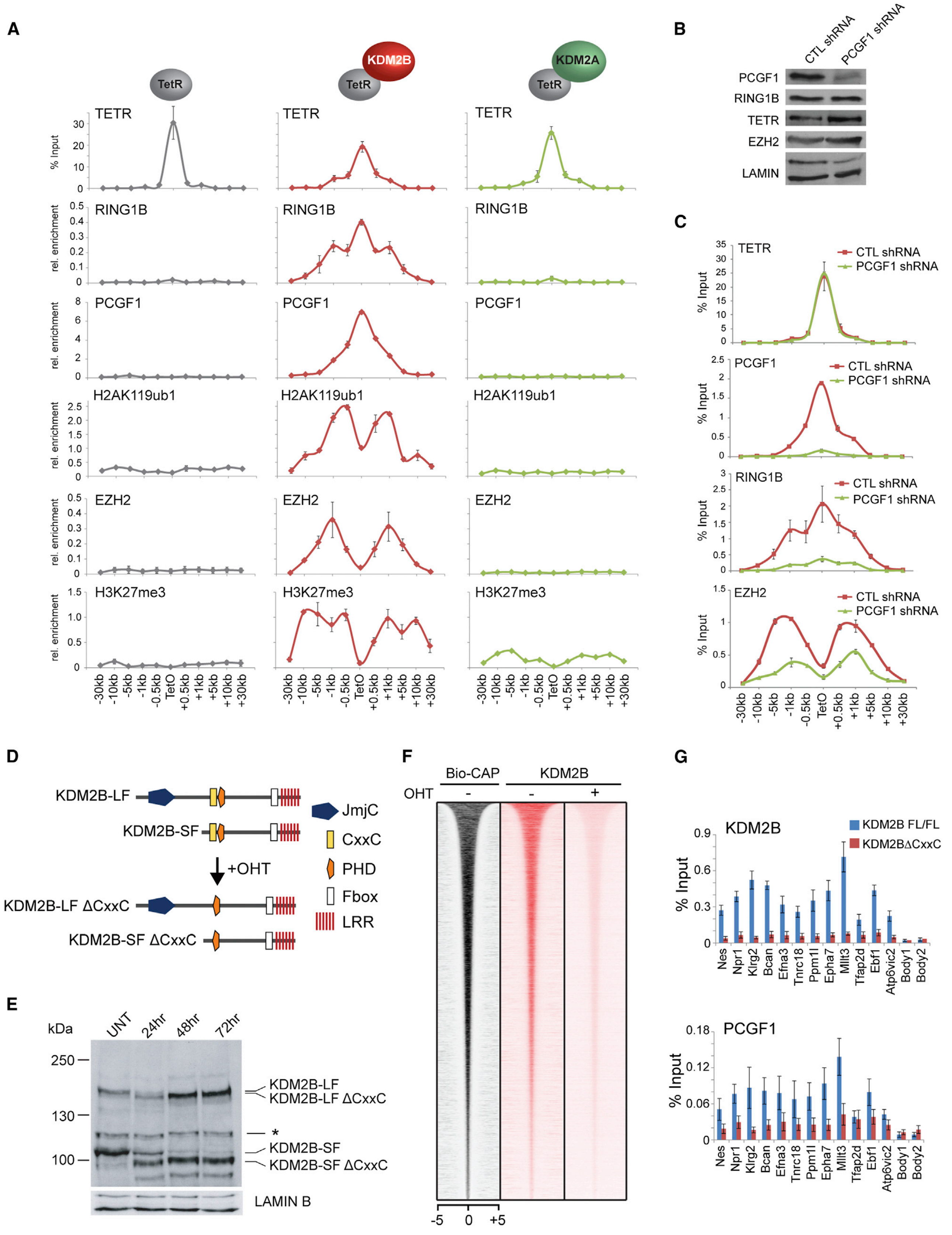

(legend on next page)

Ring1a$^{-/-}$*Ring1b*$^{fl/fl}$ system, the magnitude of RING1B and SUZ12 loss correlated well (Figure 6F), suggesting a direct relationship between PRC1 and PRC2 occupancy. Sites showing PRC2 loss also showed lower H3K27me3 levels (Figure 6I) and reduced binding of the canonical PRC1 complex component PCGF2 (Figure 6I). Interestingly, following loss of PCGF1/PRC1 targeting, genes with decreased RING1B occupancy exhibited only a very modest upregulation of average gene expression when analyzed by RNA-seq (Figures 6C and 6G), and the magnitude of RING1B loss and gene expression change showed little correlation (Figures 6H, 6I, and 6J). This supports a model whereby direct targeting of PRC1 to target sites in vivo contributes to the occupancy of PRC2, independently of large changes in gene expression.

Disruption of PCGF1/PRC1 Targeting Leads to Axial Skeletal Transformations and Embryonic Lethality in Mice

Perturbation of *Ring1a* or *Ring1b* in mice causes axial skeletal transformations (del Mar Lorente et al., 2000; Suzuki et al., 2002) due to defects in *Hox* gene expression, while deletion of *Ring1b* alone or both *Ring1a* and *Ring1b* leads to embryonic lethality (Posfai et al., 2012; Voncken et al., 2003). To understand how PCGF1/PRC1 targeting affects development, mice hemizygous for loss of the KDM2B ZF-CxxC domain were generated by crossing *Kdm2b*$^{fl/fl}$ mice to a mouse constitutively expressing Cre-recombinase. Initial observations suggested that loss of the KDM2B ZF-CxxC domain was semi-lethal as few heterozygous mice were recovered. When *Kdm2b*$^{wt/\Delta CxxC}$ were mated to wild-type mice only 20% of offspring at 10 days postnatal (dpn) were *Kdm2b*$^{wt/\Delta CxxC}$, suggesting partial haploinsufficiency (Figure 7A). To examine if the *Kdm2b*$^{wt/\Delta CxxC}$ mice exhibited homeotic transformations, skeletal preparations from newborn (n = 10) and 11 dpn (n = 2) *Kdm2b*$^{wt/\Delta CxxC}$ mice were compared to control *Kdm2b*$^{wt/wt}$ mice. All of the *Kdm2b*$^{wt/\Delta CxxC}$ heterozygous animals exhibited skeletal alterations with homeotic transformations in cervical to sacral regions (Figures 7B and 7C). Notably, seven of the ten newborn and both of the 11 dpn *Kdm2b*$^{wt/\Delta CxxC}$ mice had extra bony elements at vertebrae C7, suggesting partial transformation into T1 (Kondo and Duboule, 1999). While the second thoracic vertebra (T2) usually has a dorsal process, two of the newborn heterozygotes showed a dorsal process at T1 suggesting T1 to T2 transformation. Furthermore, dorsal processes were absent from T2 in six newborn and both 11 dpn heterozygotes, suggesting transformation of T2 to T3. Finally, two of the newborn heterozygotes showed L6-S1 transformations. Together, these homeotic phenotypes indicate posterior transformation of the vertebral column and phenocopy classical polycomb mutations (Akasaka et al., 1996; van der Lugt et al., 1994).

Attempts to generate *Kdm2b*$^{\Delta CxxC/\Delta CxxC}$ mice yielded no viable offspring. An initial examination of the embryonic defects in *Kdm2b*$^{\Delta CxxC/\Delta CxxC}$ embryos at 9 days postcoitum (dpc) suggested that development ceased at around 7 to 8 dpc in five of the embryos and two further embryos were composed only of extraembryonic tissues (Figure 7D). These phenotypes are much more severe than those previously reported for a *Kdm2b* mutant mouse (Fukuda et al., 2011). However, in this previous study, only the long form of KDM2B was disrupted, while the short form of KDM2B, which can still target the PCGF1/PRC1 complex (Figure S4) was unperturbed. Therefore, complete ablation of PCGF1/PRC1 targeting by removal of the ZF-CxxC domain from both KDM2B isoforms reveals that this activity is essential for normal development.

DISCUSSION

The co-occupancy of polycomb group proteins at target sites has largely been viewed in the context of a hierarchical recruitment model (Cao et al., 2002; Wang et al., 2004b). Although this mechanism is clearly relevant for RING1B accumulation on chromatin, the singularity of the hierarchical recruitment pathway has recently been challenged by a series of observations in mammals demonstrating that PRC1 occupancy and H2AK119ub1 can be achieved in the absence of PRC2 (Pasini et al., 2007; Schoeftner et al., 2006; Tavares et al., 2012) and observations in *Drosophila* suggesting that H3K27me3 is not sufficient to recruit PRC1 complexes (Schwartz et al., 2006). We now unexpectedly discover that variant, but not canonical, PRC1 complex occupancy leads to binding of PRC2 and placement of H3K27me3 in an H2AK119ub1-dependent manner, with deletion of the catalytic core of PRC1 in mouse ESCs resulting in a dramatic reduction in PRC2 and H3K27me3 at target sites. Furthermore, KDM2B-mediated targeting of the variant PCGF1/PRC1 complex to CpG islands is required for normal PRC2 levels at a subset of target sites and failure to target PCGF1/PRC1 results in polycomb phenotypes and embryonic lethality in mice. This provides an alternative to the hierarchical recruitment mechanism, effectively demonstrating that PRC1 complexes are not simply subservient readers of PRC2 activity but can instead be actively recruited to target sites and act as central players in polycomb domain formation.

Figure 5. KDM2B Targets the PCGF1/PRC1 Variant Complex Leading to PRC2 Recruitment and Formation of a Polycomb Domain and a Novel System to Ablate KDM2B-Mediated Targeting of PCGF1/PRC1 to Chromatin

(A) ChIP analysis for TetR, RING1B, PCGF1, H2AK119ub1, EZH2, and H3K27me3 across the TetO containing locus in lines expressing TetR, TetR-KDM2B, and TetR-KDM2A. ChIP experiments were performed at least in biological duplicate with error bars showing SEM.

(B) Western blot analysis of PRC1 and PRC2 protein levels after knockdown of PCGF1 in the TetR-KDM2B fusion line.

(C) ChIP analysis for TetR, PCGF1, RING1B, and EZH2 following PCGF1 knockdown in the TetR-KDM2B fusion line. ChIP experiments were performed at least in biological duplicate with error bars showing SEM.

(D) A schematic illustrating tamoxifen (OHT)-dependent removal of the ZF-CxxC domain from both KDM2B isoforms.

(E) Western blot analysis of the *Kdm2b*$^{fl/fl}$ cell line following an OHT treatment time course. (*) is a nonspecific cross reactive band.

(F) A heat map covering a 10 kb region centered over CpG islands showing KDM2B ChIP-seq in the *Kdm2b*$^{fl/fl}$ cells prior to (−) and after 72 hr (+) OHT treatment.

(G) ChIP analysis of KDM2B and PCGF1 at gene-associated CpG islands and gene body regions. ChIP experiments were performed in biological triplicate with error bars showing SEM. See also Figures S4 and S5.

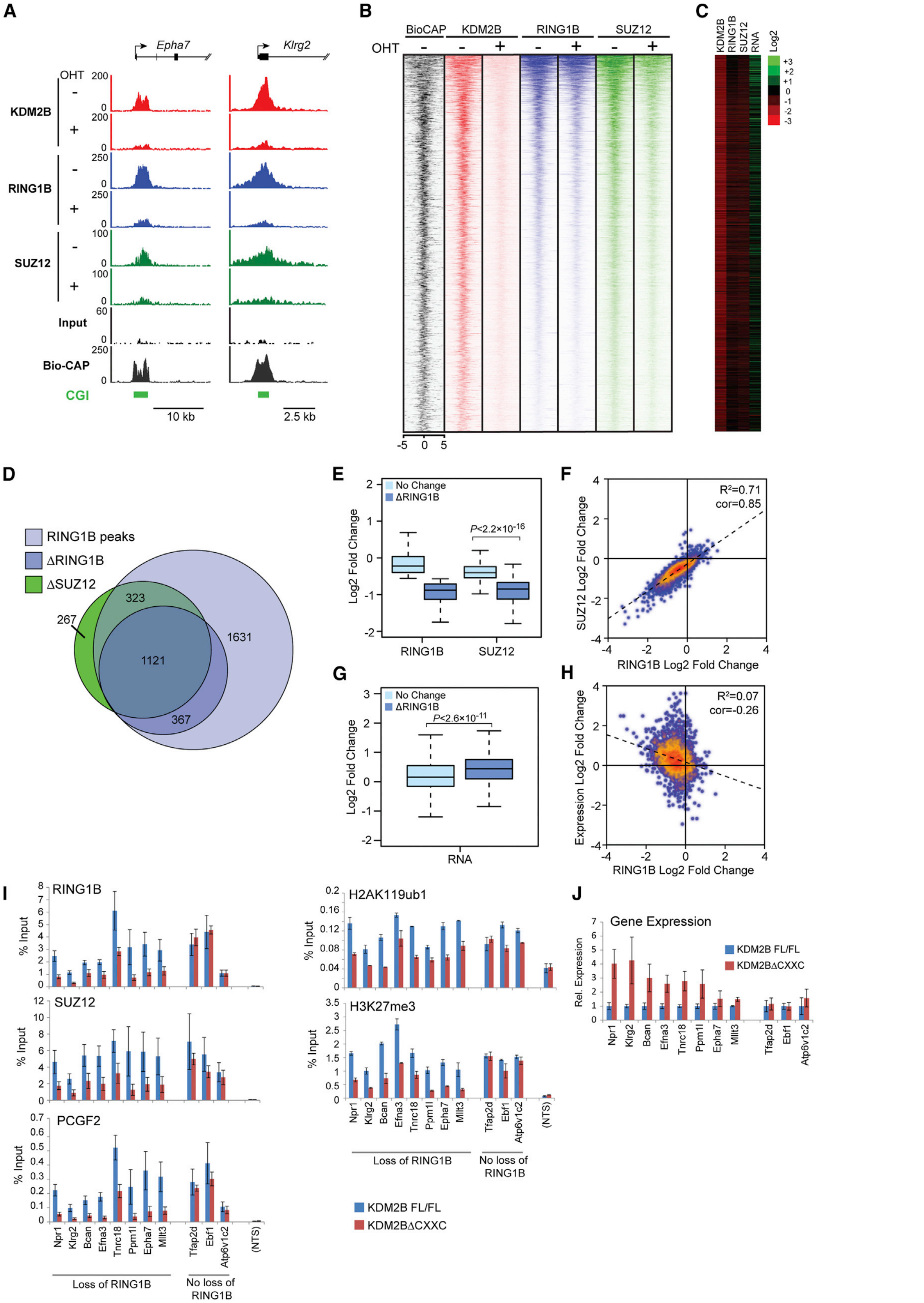

(legend on next page)

H2AK119ub1 levels are unaffected in ESCs lacking PRC2 (Schoeftner et al., 2006; Tavares et al., 2012) and disruption of canonical PRC1-complex-specific subunits in *Drosophila* cell culture does not significantly affect H2A ubiquitylation (Lagarou et al., 2008). This is consistent with the failure of canonical PRC1 complexes to place H2AK119ub1 in tethering experiments (Figure 1). Therefore, despite their competence to catalyze H2AK119ub1 in vitro (Gao et al., 2012; Tavares et al., 2012), canonical complexes do not seem to play a major role in H2AK119ub1 deposition in vivo. This apparent discrepancy may result from a subunit specific to canonical PRC1 complexes that limits RING1A/B E3 ligase activity in cells, as a minimal catalytic fusion of PCGF4 and RING1B that does not interact with other canonical PRC1 complex proteins was competent to catalyze H2AK119ub1 (Figure 3), whereas the intact canonical PCGF4/PRC1 complex appeared largely inactive (Figure 1). A candidate for this inhibitory activity may be the vertebrate polyhomeotic orthologs (PHC1/2/3), which are specific to canonical PRC1 complexes. PHC proteins have been shown to self-associate via their sterile alpha motif (SAM) to organize polycomb domains in vivo (Isono et al., 2013; Kim et al., 2002). It is tempting to speculate that the hierarchical recruitment pathway may largely function to drive PHC proteins to sites that have already acquired a polycomb domain and impose further alterations in chromatin structure.

The unexpected discovery that H2AK119ub1 plays a critical role in PRC2 occupancy and H3K27me3 at target sites appears at odds with observations in mouse ESCs lacking RING1B where PRC2 function appeared less affected (Eskeland et al., 2010; Leeb et al., 2010; van der Stoop et al., 2008). However, RING1B null cells express RING1A and retain significant levels of H2AK119ub1 (Eskeland et al., 2010), suggesting that the relative level of H2AK119ub1 may be important for PRC2 occupancy. Importantly, PRC1 activity appears to drive PRC2 occupancy in other nonstereotypical examples of polycomb domain formation, suggesting this activity is not limited by genomic location (Cooper et al., 2014). A future challenge remains to understand the molecular determinants that link H2AK119ub1 and PRC2 binding.

It is a commonly held view that mammalian polycomb proteins, under the guidance of site-specific DNA binding transcription factors or lncRNAs, are targeted to specific genes where they direct transcriptional repression (discussed in Simon and Kingston, 2013). In stark contrast to site-specific targeting factors, KDM2B binds broadly to promoter-associated CpG islands through its nonmethylated DNA-binding activity, occupying genes covering the complete expression spectrum (Farcas et al., 2012; He et al., 2013; Wu et al., 2013). Given the capacity of KDM2B to bind CpG islands, target the PCGF1/PRC1 variant complex, and initiate polycomb domains de novo, it is surprising that only a subset of the most repressed CpG island-associated genes accumulate polycomb proteins. A possible explanation for this somewhat paradoxical observation could be that counteracting chromatin features associated with transcription are sufficient to inhibit polycomb protein activity and that KDM2B/PCGF1/PRC1 occupancy functions mainly as a sampling module to identify sites that lack transcription and facilitate initiation of a polycomb domain (Klose et al., 2013; Ku et al., 2008; Lynch et al., 2012; Voigt et al., 2013). Viewed in this context, one would predict that polycomb domain formation would not directly drive gene repression, but may instead function to limit stochastic or inappropriate reactivation of genes that have already been transcriptionally silenced. Consistent with these ideas, it was recently demonstrated through an elegant set of kinetic experiments in mammalian cell culture systems that polycomb chromatin modifications are mainly acquired at target sites after transcriptional silencing has been achieved (Hosogane et al., 2013; Yuan et al., 2012). This agrees with the more general observation that loss of polycomb proteins in ESCs leads to reactivation of only a subset of all genes that are heavily occupied by polycomb proteins (Leeb et al., 2010). In agreement with these general principles, loss of PCGF1/PRC1 targeting and its capacity to form polycomb domains did not lead to extensive gene reactivation (Figure 6).

It is clear that variant PRC1 complexes can drive de novo PRC2 occupancy (Figure 1), and PRC1 is required for normal polycomb domain formation (Figure 4). A variant PRC1 complex-driven sampling model could provide a simple and flexible solution for polycomb domain initiation and formation, but it should be made clear that our observations do not exclude potential contributions from PRC1-independent PRC2 targeting events. Following PRC1 deletion, there was a reduction, but

Figure 6. Failure to Target the PCGF1/PRC1 Complex Leads to a Loss of H2AK119ub1, PRC2, and H3K27me3

(A) Snapshots of ChIP-seq traces for KDM2B, RING1B, and SUZ12 in the *Kdm2b*$^{fl/fl}$ cells prior to (−OHT) and after 72 hr (+OHT) of tamoxifen treatment at the *Epha7* and *Klrg2* genes.

(B) Heat map analysis of RING1B peaks (n = 3,488), showing ChIP-seq data for KDM2B, RING1B, and SUZ12 covering a 10 kb region centered over the RING1B peak −OHT and after 72 hr +OHT.

(C) Log2 fold changes in normalized read counts comparing the ChIP-seq and RNA-seq signal −OHT and after 72 hr +OHT.

(D) A Venn diagram showing all RING1B peaks (light blue), intersected with RING1B or SUZ12 peaks that have a greater than 1.5-fold reduction in RING1B/SUZ12 occupancy after 72 hr +OHT treatment (ΔRING1B [dark blue] and ΔSUZ12 [green]).

(E) A box and whisker plot indicating the log2 fold change in RING1B and SUZ12 occupancy at sites that have RING1B changes of greater than 1.5-fold (ΔRING1B) or less than 1.5-fold (No Change) following 72 hr +OHT treatment. The significance of the changes in SUZ12 occupancy at these sites is indicated above the plot.

(F) A scatter plot comparing the log2 fold change of RING1B and SUZ12 at RING1B sites in the *Kdm2b*$^{fl/fl}$ cells −OHT and after 72 hr +OHT.

(G) A box and whisker plot indicating log2 fold change in gene expression at sites described in (E).

(H) A scatter plot comparing the log2 fold change of gene expression to the fold change in RING1B occupancy at sites that show RING1B alterations.

(I) ChIP analysis for polycomb factors and modifications at regions showing loss of RING1B, no significant loss of RING1B, and a nontarget site (NTS). All ChIP experiments were performed in biological triplicate with error bars showing SEM.

(J) Gene expression analysis for the target genes analyzed by ChIP in (I). RT-PCR was performed in biological triplicate. Error bars show SEM.

Genotype	*Kdm2b* WT/WT	*Kdm2b* WT/ΔCxxC
Number	22	5

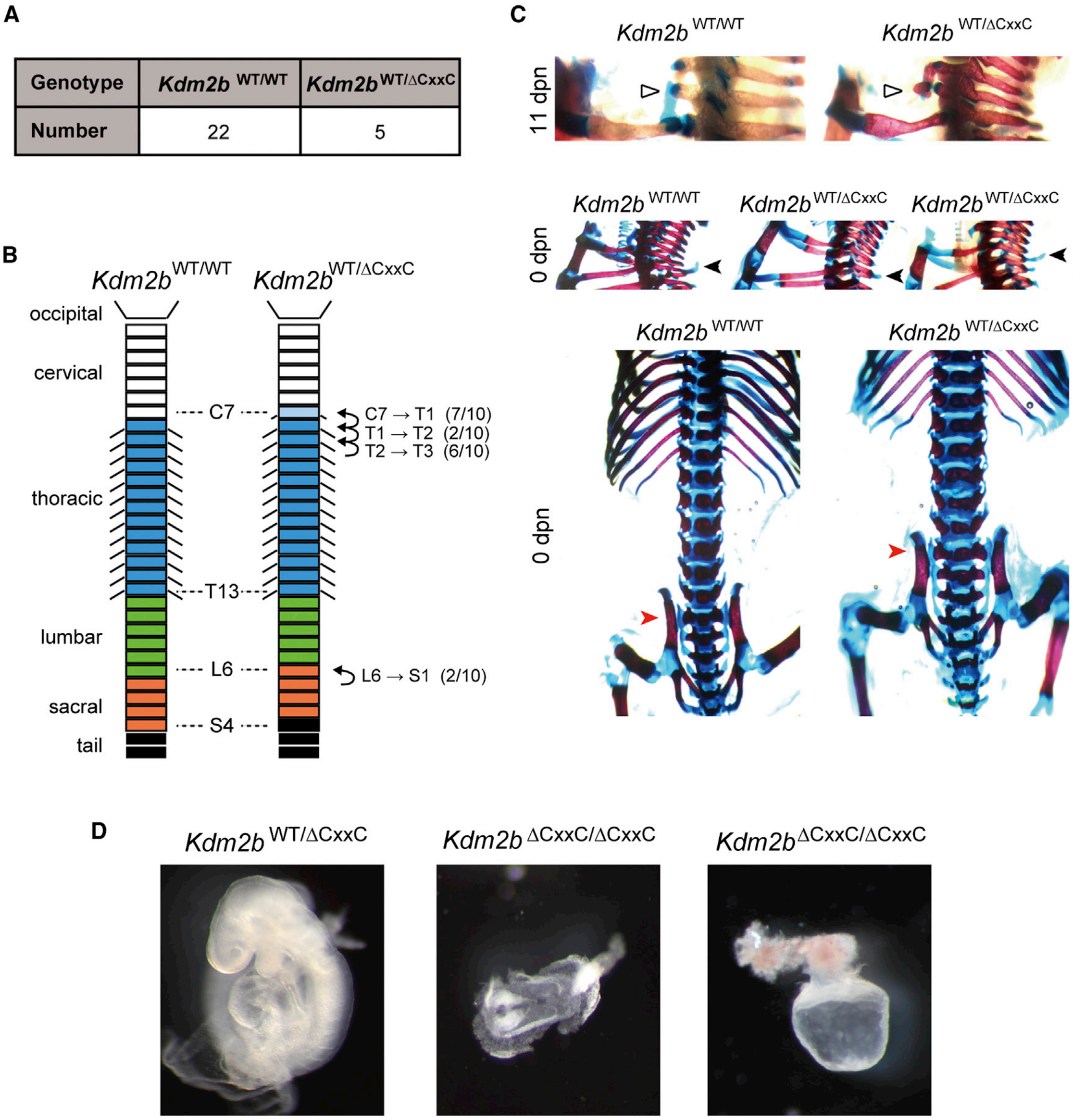

Figure 7. Failure to Target PCGF1/PRC1 Results in Homeotic Phenotypes and Embryonic Lethality

(A) *Kdm2b*$^{wt/\Delta CxxC}$ mice were mated with wild-type mice and at 11 day postnatal (dpn) offspring were genotyped. Results are summarized in a table.

(B) A schematic summarizing the homeotic phenotypes observed in newborn *Kdm2b*$^{wt/\Delta CxxC}$ mice (n = 10) with the normal wild-type vertebrae organization shown for comparison. The numbers in parentheses indicate the frequency of individual transformations.

(C) Examples of the vertebral column in wild-type and *Kdm2b*$^{wt/\Delta CxxC}$ mice showing homeotic phenotypes. Top: 11 dpn heterozygotes that have additional ossification at the C7 position indicating partial posteriorization (white triangles). Center: comparison of newborn wild-type and newborn *Kdm2b*$^{wt/\Delta CxxC}$ mice missing dorsal processes or that have the position of the process repositioned to the anterior (black arrowhead). Bottom: 6th lumbar vertebral column transformed to sacrum in *Kdm2b*$^{wt/\Delta CxxC}$ mice (red arrowhead).

(D) *Kdm2b* homozygous null embryos recovered at 9 dpc exhibited severe developmental delay (n = 5) or in some cases only extraembryonic development (n = 2) (representative examples are shown in center and right, respectively). A heterozygote sibling is shown to indicate expected development at this stage (left).

not a complete loss, of PRC2 and H3K27me3 (Figure 4). While residual PRC1 binding or epigenetic maintenance of preexisting PRC2 could be responsible for this, previously reported PRC1-independent targeting mechanisms could also play a central role in remaining PRC2 occupancy (Simon and Kingston, 2013). Viewed in this light, it remains possible that PRC1 and PRC2 are recruited to target sites independently and then function to mutually sustain and stabilize their respective binding. A better understanding of the molecular mechanisms that underpin polycomb protein targeting will help to further define the relationship between PRC1 and PRC2 on chromatin.

Cancer-exome-sequencing endeavours have recently revealed that core components of the KDM2B/PRC1 complex, including KDM2B itself and BCOR/L1, are frequently mutated in a range of cancers, particularly leukemias (Brookes et al., 2012; Grossmann et al., 2011; Pasqualucci et al., 2011; Zhang et al., 2012). Given the essential nature of polycomb protein function in vertebrate development and its implication in human pathology, our fundamental discovery that PRC1 activity plays an important role in normal PRC2 occupancy provides an unexpected new perspective on the principles that underpin polycomb domain formation. Furthermore, toward addressing the central yet poorly understood question of defining how polycomb sites are established in vivo, we provide evidence that, at least in some cases, there may exist a relatively simple molecular chain of events whereby KDM2B-mediated recognition of nonmethylated DNA at receptive CpG islands leads to recruitment of PRC1 and deposition of H2AK119ub1 that ultimately translates into occupancy of PRC2 necessary for normal placement of H3K27me3.

EXPERIMENTAL PROCEDURES

Generation of the De Novo Targeting System

A TetO array comprising 14 TetO sites interspersed by random CpG-free 21 bp sequences was seamlessly recombineered into BAC RP11 419E6. Tol2 sequence elements were then recombineered into the plasmid backbone portion of the BAC, together with a neomycin resistance cassette. Using Lipofectamine 2000 (Life Technologies) the TetO BAC was cotransfected with a Tol2 transposase expression plasmid into E14 mouse ESCs, and stable G418 (400 μg/ml) resistant clones were obtained. A PCR screen was used to identify a clone exhibiting Tol2-mediated integration and splinkerette PCR was used to map the precise BAC integration site on mouse chromosome 8.

The TetR coding sequence was inserted into a modified pCAG-IRES-Puro mammalian expression plasmid, between the coding sequence for an N-terminal FLAG STREPx2 (FS2) tag and a 3′ ligation-independent cloning (LIC) site. The resultant plasmid was named pCAGFS2TetR. Coding sequences for proteins of interest were inserted into pCAGFS2TetR by LIC cloning. All plasmids were transfected into the TetO BAC-containing ESCs and stable clones expressing TetR fusion proteins were obtained by selection with puromycin (1 μg/ml). TetR fusion proteins were detected in ChIP experiments using an FS2-specific antibody. Other details of the TetR targeting system are described in Extended Experimental Procedures.

Chromatin Immunoprecipitation

ChIP was performed as described previously (Farcas et al., 2012), with minor modifications. Briefly, for nonhistone ChIP cells were fixed for 1 hr in 2 mM EGS, followed by 15 min in 1% formaldehyde, while for histone modification ChIP cells were fixed for 10 min in 1% formaldehyde alone.

Cells were sequentially lysed and sonication was performed to produce fragments of approximately 0.5–1 kb. Immunoprecipitation was performed overnight at 4°C with approximately 3 μg of antibody. Antibody-bound chromatin was isolated on protein A beads, washed extensively and purified as described in Extended Experimental Procedures.

Gene Expression Analysis

For RNA-seq analysis, polyA+ RNA was purified from total RNA and then a directional library was prepared using the NEBNext Ultra Directional RNA library Prep Kit for Illumina (NEB, Ipswich, MA).

ACCESSION NUMBERS

The Geo accession number for the ChIP-seq and RNA-seq data reported in this paper is GSE55698.

SUPPLEMENTAL INFORMATION

Supplemental Information includes Extended Experimental Procedures and five figures and can be found with this article online at http://dx.doi.org/10.1016/j.cell.2014.05.004.

AUTHOR CONTRIBUTIONS

N.P.B. designed and created TetR tethering system. A.M.F. performed affinity purifications and generated PCGF1 antibody. N.P.B. and A.M.F. generated individual TetR-fusion cell lines, and performed all ChIP and RT-PCR experiments. T.K. generated the *Kdm2b*$^{fl/fl}$ mouse and ESCs and performed mouse phenotype characterization. N.P.B, A.M.F and T.K. prepared the manuscript.

ACKNOWLEDGMENTS

We thank Dr. Tom Milne for comments and suggestions, Marie-Laetitia Thezenas and Dr. Rebecca Konietzny for mass spectrometry expertise. This study was funded by the Wellcome Trust (WT0834922 and WT081385), CRUK (C28585/A10839), NIHR, EMBO, Lister Institute of Preventative Medicine, RIKEN, MEXT, and JST CREST. The WTCHG Genomics Facility carried out massively parallel sequencing.

Received: November 20, 2013
Revised: March 10, 2014
Accepted: May 2, 2014
Published: May 22, 2014

REFERENCES

Akasaka, T., Kanno, M., Balling, R., Mieza, M.A., Taniguchi, M., and Koseki, H. (1996). A role for mel-18, a Polycomb group-related vertebrate gene, during theanteroposterior specification of the axial skeleton. Development *122*, 1513–1522.

Arnold, P., Schöler, A., Pachkov, M., Balwierz, P.J., Jørgensen, H., Stadler, M.B., van Nimwegen, E., and Schübeler, D. (2013). Modeling of epigenome dynamics identifies transcription factors that mediate Polycomb targeting. Genome Res. *23*, 60–73.

Bentley, M.L., Corn, J.E., Dong, K.C., Phung, Q., Cheung, T.K., and Cochran, A.G. (2011). Recognition of UbcH5c and the nucleosome by the Bmi1/Ring1b ubiquitin ligase complex. EMBO J. *30*, 3285–3297.

Blackledge, N.P., Zhou, J.C., Tolstorukov, M.Y., Farcas, A.M., Park, P.J., and Klose, R.J. (2010). CpG islands recruit a histone H3 lysine 36 demethylase. Mol. Cell *38*, 179–190.

Boyer, L.A., Plath, K., Zeitlinger, J., Brambrink, T., Medeiros, L.A., Lee, T.I., Levine, S.S., Wernig, M., Tajonar, A., Ray, M.K., et al. (2006). Polycomb complexes repress developmental regulators in murine embryonic stem cells. Nature *441*, 349–353.

Bracken, A.P., and Helin, K. (2009). Polycomb group proteins: navigators of lineage pathways led astray in cancer. Nat. Rev. Cancer *9*, 773–784.

Bracken, A.P., Dietrich, N., Pasini, D., Hansen, K.H., and Helin, K. (2006). Genome-wide mapping of Polycomb target genes unravels their roles in cell fate transitions. Genes Dev. *20*, 1123–1136.

Brookes, E., de Santiago, I., Hebenstreit, D., Morris, K.J., Carroll, T., Xie, S.Q., Stock, J.K., Heidemann, M., Eick, D., Nozaki, N., et al. (2012). Polycomb associates genome-wide with a specific RNA polymerase II variant, and regulates metabolic genes in ESCs. Cell Stem Cell *10*, 157–170.

Buchwald, G., van der Stoop, P., Weichenrieder, O., Perrakis, A., van Lohuizen, M., and Sixma, T.K. (2006). Structure and E3-ligase activity of the Ring-Ring complex of polycomb proteins Bmi1 and Ring1b. EMBO J. *25*, 2465–2474.

Cao, R., and Zhang, Y. (2004). SUZ12 is required for both the histone methyltransferase activity and the silencing function of the EED-EZH2 complex. Mol. Cell *15*, 57–67.

Cao, R., Wang, L., Wang, H., Xia, L., Erdjument-Bromage, H., Tempst, P., Jones, R.S., and Zhang, Y. (2002). Role of histone H3 lysine 27 methylation in Polycomb-group silencing. Science *298*, 1039–1043.

Chamberlain, S.J., Yee, D., and Magnuson, T. (2008). Polycomb repressive complex 2 is dispensable for maintenance of embryonic stem cell pluripotency. Stem Cells *26*, 1496–1505.

Cooper, S., Dienstbier, M., Hassan, R., Schermelleh, L., Sharif, J., Blackledge, N.P., De Marco, V., Elderkin, S., Koseki, H., Klose, R., et al. (2014). Targeting Polycomb to Pericentric Heterochromatin in Embryonic Stem Cells Reveals a Role for H2AK119u1 in PRC2 Recruitment. Cell Rep. *7*. Published online May 22, 2014. http://dx.doi.org/10.1016/j.celrep.2014.04.012.

Czermin, B., Melfi, R., McCabe, D., Seitz, V., Imhof, A., and Pirrotta, V. (2002). Drosophila enhancer of Zeste/ESC complexes have a histone H3 methyltransferase activity that marks chromosomal Polycomb sites. Cell *111*, 185–196.

de Napoles, M., Mermoud, J.E., Wakao, R., Tang, Y.A., Endoh, M., Appanah, R., Nesterova, T.B., Silva, J., Otte, A.P., Vidal, M., et al. (2004). Polycomb group proteins Ring1A/B link ubiquitylation of histone H2A to heritable gene silencing and X inactivation. Dev. Cell *7*, 663–676.

del Mar Lorente, M., Marcos-Gutiérrez, C., Pérez, C., Schoorlemmer, J., Ramírez, A., Magin, T., and Vidal, M. (2000). Loss- and gain-of-function mutations show a polycomb group function for Ring1A in mice. Development *127*, 5093–5100.

Dietrich, N., Lerdrup, M., Landt, E., Agrawal-Singh, S., Bak, M., Tommerup, N., Rappsilber, J., Södersten, E., and Hansen, K. (2012). REST-mediated recruitment of polycomb repressor complexes in mammalian cells. PLoS Genet. *8*, e1002494.

Endoh, M., Endo, T.A., Endoh, T., Fujimura, Y., Ohara, O., Toyoda, T., Otte, A.P., Okano, M., Brockdorff, N., Vidal, M., and Koseki, H. (2008). Polycomb group proteins Ring1A/B are functionally linked to the core transcriptional regulatory circuitry to maintain ES cell identity. Development *135*, 1513–1524.

Eskeland, R., Leeb, M., Grimes, G.R., Kress, C., Boyle, S., Sproul, D., Gilbert, N., Fan, Y., Skoultchi, A.I., Wutz, A., and Bickmore, W.A. (2010). Ring1B compacts chromatin structure and represses gene expression independent of histone ubiquitination. Mol. Cell *38*, 452–464.

Farcas, A.M., Blackledge, N.P., Sudbery, I., Long, H.K., McGouran, J.F., Rose, N.R., Lee, S., Sims, D., Cerase, A., Sheahan, T.W., et al. (2012). KDM2B links the Polycomb Repressive Complex 1 (PRC1) to recognition of CpG islands. Elife *1*, e00205.

Fukuda, T., Tokunaga, A., Sakamoto, R., and Yoshida, N. (2011). Fbxl10/Kdm2b deficiency accelerates neural progenitor cell death and leads to exencephaly. Mol. Cell. Neurosci. *46*, 614–624.

Gao, Z., Zhang, J., Bonasio, R., Strino, F., Sawai, A., Parisi, F., Kluger, Y., and Reinberg, D. (2012). PCGF homologs, CBX proteins, and RYBP define functionally distinct PRC1 family complexes. Mol. Cell *45*, 344–356.

Gearhart, M.D., Corcoran, C.M., Wamstad, J.A., and Bardwell, V.J. (2006). Polycomb group and SCF ubiquitin ligases are found in a novel BCOR complex that is recruited to BCL6 targets. Mol. Cell. Biol. *26*, 6880–6889.

Grossmann, V., Tiacci, E., Holmes, A.B., Kohlmann, A., Martelli, M.P., Kern, W., Spanhol-Rosseto, A., Klein, H.U., Dugas, M., Schindela, S., et al. (2011). Whole-exome sequencing identifies somatic mutations of BCOR in acute myeloid leukemia with normal karyotype. Blood *118*, 6153–6163.

Hansen, K.H., Bracken, A.P., Pasini, D., Dietrich, N., Gehani, S.S., Monrad, A., Rappsilber, J., Lerdrup, M., and Helin, K. (2008). A model for transmission of the H3K27me3 epigenetic mark. Nat. Cell Biol. *10*, 1291–1300.

He, J., Shen, L., Wan, M., Taranova, O., Wu, H., and Zhang, Y. (2013). Kdm2b maintains murine embryonic stem cell status by recruiting PRC1 complex to CpG islands of developmental genes. Nat. Cell Biol. *15*, 373–384.

Hosogane, M., Funayama, R., Nishida, Y., Nagashima, T., and Nakayama, K. (2013). Ras-induced changes in H3K27me3 occur after those in transcriptional activity. PLoS Genet. *9*, e1003698.

Isono, K., Endo, T.A., Ku, M., Yamada, D., Suzuki, R., Sharif, J., Ishikura, T., Toyoda, T., Bernstein, B.E., and Koseki, H. (2013). SAM domain polymerization links subnuclear clustering of PRC1 to gene silencing. Dev. Cell *26*, 565–577.

Kaneko, S., Bonasio, R., Saldaña-Meyer, R., Yoshida, T., Son, J., Nishino, K., Umezawa, A., and Reinberg, D. (2014). Interactions between JARID2 and noncoding RNAs regulate PRC2 recruitment to chromatin. Mol. Cell *53*, 290–300.

Ketel, C.S., Andersen, E.F., Vargas, M.L., Suh, J., Strome, S., and Simon, J.A. (2005). Subunit contributions to histone methyltransferase activities of fly and worm polycomb group complexes. Mol. Cell. Biol. *25*, 6857–6868.

Kim, C.A., Gingery, M., Pilpa, R.M., and Bowie, J.U. (2002). The SAM domain of polyhomeotic forms a helical polymer. Nat. Struct. Biol. *9*, 453–457.

Klose, R.J., Cooper, S., Farcas, A.M., Blackledge, N.P., and Brockdorff, N. (2013). Chromatin sampling—an emerging perspective on targeting polycomb repressor proteins. PLoS Genet. *9*, e1003717.

Kondo, T., and Duboule, D. (1999). Breaking colinearity in the mouse HoxD complex. Cell *97*, 407–417.

Ku, M., Koche, R.P., Rheinbay, E., Mendenhall, E.M., Endoh, M., Mikkelsen, T.S., Presser, A., Nusbaum, C., Xie, X., Chi, A.S., et al. (2008). Genomewide analysis of PRC1 and PRC2 occupancy identifies two classes of bivalent domains. PLoS Genet. *4*, e1000242.

Kuzmichev, A., Nishioka, K., Erdjument-Bromage, H., Tempst, P., and Reinberg, D. (2002). Histone methyltransferase activity associated with a human multiprotein complex containing the Enhancer of Zeste protein. Genes Dev. *16*, 2893–2905.

Lagarou, A., Mohd-Sarip, A., Moshkin, Y.M., Chalkley, G.E., Bezstarosti, K., Demmers, J.A., and Verrijzer, C.P. (2008). dKDM2 couples histone H2A ubiquitylation to histone H3 demethylation during Polycomb group silencing. Genes Dev. *22*, 2799–2810.

Leeb, M., Pasini, D., Novatchkova, M., Jaritz, M., Helin, K., and Wutz, A. (2010). Polycomb complexes act redundantly to repress genomic repeats and genes. Genes Dev. *24*, 265–276.

Levine, S.S., Weiss, A., Erdjument-Bromage, H., Shao, Z., Tempst, P., and Kingston, R.E. (2002). The core of the polycomb repressive complex is compositionally and functionally conserved in flies and humans. Mol. Cell. Biol. *22*, 6070–6078.

Li, Z., Cao, R., Wang, M., Myers, M.P., Zhang, Y., and Xu, R.M. (2006). Structure of a Bmi-1-Ring1B polycomb group ubiquitin ligase complex. J. Biol. Chem. *281*, 20643–20649.

Long, H.K., Blackledge, N.P., and Klose, R.J. (2013). ZF-CxxC domain-containing proteins, CpG islands and the chromatin connection. Biochem. Soc. Trans. *41*, 727–740.

Lynch, M.D., Smith, A.J., De Gobbi, M., Flenley, M., Hughes, J.R., Vernimmen, D., Ayyub, H., Sharpe, J.A., Sloane-Stanley, J.A., Sutherland, L., et al. (2012). An interspecies analysis reveals a key role for unmethylated CpG dinucleotides in vertebrate Polycomb complex recruitment. EMBO J. *31*, 317–329.

Margueron, R., Justin, N., Ohno, K., Sharpe, M.L., Son, J., Drury, W.J., 3rd, Voigt, P., Martin, S.R., Taylor, W.R., De Marco, V., et al. (2009). Role of the polycomb protein EED in the propagation of repressive histone marks. Nature *461*, 762–767.

Min, J., Zhang, Y., and Xu, R.M. (2003). Structural basis for specific binding of Polycomb chromodomain to histone H3 methylated at Lys 27. Genes Dev. *17*, 1823–1828.

Müller, J., Hart, C.M., Francis, N.J., Vargas, M.L., Sengupta, A., Wild, B., Miller, E.L., O'Connor, M.B., Kingston, R.E., and Simon, J.A. (2002). Histone methyltransferase activity of a Drosophila Polycomb group repressor complex. Cell *111*, 197–208.

Ogawa, H., Ishiguro, K., Gaubatz, S., Livingston, D.M., and Nakatani, Y. (2002). A complex with chromatin modifiers that occupies E2F- and Myc-responsive genes in G0 cells. Science *296*, 1132–1136.

Papp, B., and Müller, J. (2006). Histone trimethylation and the maintenance of transcriptional ON and OFF states by trxG and PcG proteins. Genes Dev. *20*, 2041–2054.

Pasini, D., Bracken, A.P., Jensen, M.R., Lazzerini Denchi, E., and Helin, K. (2004). Suz12 is essential for mouse development and for EZH2 histone methyltransferase activity. EMBO J. *23*, 4061–4071.

Pasini, D., Bracken, A.P., Hansen, J.B., Capillo, M., and Helin, K. (2007). The polycomb group protein Suz12 is required for embryonic stem cell differentiation. Mol. Cell. Biol. *27*, 3769–3779.

Pasqualucci, L., Trifonov, V., Fabbri, G., Ma, J., Rossi, D., Chiarenza, A., Wells, V.A., Grunn, A., Messina, M., Elliot, O., et al. (2011). Analysis of the coding genome of diffuse large B-cell lymphoma. Nat. Genet. *43*, 830–837.

Posfai, E., Kunzmann, R., Brochard, V., Salvaing, J., Cabuy, E., Roloff, T.C., Liu, Z., Tardat, M., van Lohuizen, M., Vidal, M., et al. (2012). Polycomb function during oogenesis is required for mouse embryonic development. Genes Dev. *26*, 920–932.

Sánchez, C., Sánchez, I., Demmers, J.A., Rodriguez, P., Strouboulis, J., and Vidal, M. (2007). Proteomics analysis of Ring1B/Rnf2 interactors identifies a novel complex with the Fbxl10/Jhdm1B histone demethylase and the Bcl6 interacting corepressor. Mol. Cell. Proteomics *6*, 820–834.

Schoeftner, S., Sengupta, A.K., Kubicek, S., Mechtler, K., Spahn, L., Koseki, H., Jenuwein, T., and Wutz, A. (2006). Recruitment of PRC1 function at the initiation of X inactivation independent of PRC2 and silencing. EMBO J. *25*, 3110–3122.

Schwartz, Y.B., and Pirrotta, V. (2013). A new world of Polycombs: unexpected partnerships and emerging functions. Nat. Rev. Genet. *14*, 853–864.

Schwartz, Y.B., Kahn, T.G., Nix, D.A., Li, X.Y., Bourgon, R., Biggin, M., and Pirrotta, V. (2006). Genome-wide analysis of Polycomb targets in Drosophila melanogaster. Nat. Genet. *38*, 700–705.

Simon, J.A., and Kingston, R.E. (2013). Occupying chromatin: Polycomb mechanisms for getting to genomic targets, stopping transcriptional traffic, and staying put. Mol. Cell *49*, 808–824.

Suzuki, M., Mizutani-Koseki, Y., Fujimura, Y., Miyagishima, H., Kaneko, T., Takada, Y., Akasaka, T., Tanzawa, H., Takihara, Y., Nakano, M., et al. (2002). Involvement of the Polycomb-group gene Ring1B in the specification of the anterior-posterior axis in mice. Development *129*, 4171–4183.

Tavares, L., Dimitrova, E., Oxley, D., Webster, J., Poot, R., Demmers, J., Bezstarosti, K., Taylor, S., Ura, H., Koide, H., et al. (2012). RYBP-PRC1 complexes mediate H2A ubiquitylation at polycomb target sites independently of PRC2 and H3K27me3. Cell *148*, 664–678.

Tolhuis, B., de Wit, E., Muijrers, I., Teunissen, H., Talhout, W., van Steensel, B., and van Lohuizen, M. (2006). Genome-wide profiling of PRC1 and PRC2 Polycomb chromatin binding in Drosophila melanogaster. Nat. Genet. *38*, 694–699.

van der Lugt, N.M., Domen, J., Linders, K., van Roon, M., Robanus-Maandag, E., te Riele, H., van der Valk, M., Deschamps, J., Sofroniew, M., van Lohuizen, M., et al. (1994). Posterior transformation, neurological abnormalities, and severe hematopoietic defects in mice with a targeted deletion of the bmi-1 proto-oncogene. Genes Dev. *8*, 757–769.

van der Stoop, P., Boutsma, E.A., Hulsman, D., Noback, S., Heimerikx, M., Kerkhoven, R.M., Voncken, J.W., Wessels, L.F., and van Lohuizen, M. (2008). Ubiquitin E3 ligase Ring1b/Rnf2 of polycomb repressive complex 1 contributes to stable maintenance of mouse embryonic stem cells. PLoS ONE *3*, e2235.

Voigt, P., Tee, W.W., and Reinberg, D. (2013). A double take on bivalent promoters. Genes Dev. *27*, 1318–1338.

Voncken, J.W., Roelen, B.A., Roefs, M., de Vries, S., Verhoeven, E., Marino, S., Deschamps, J., and van Lohuizen, M. (2003). Rnf2 (Ring1b) deficiency causes gastrulation arrest and cell cycle inhibition. Proc. Natl. Acad. Sci. USA *100*, 2468–2473.

Wang, H., Wang, L., Erdjument-Bromage, H., Vidal, M., Tempst, P., Jones, R.S., and Zhang, Y. (2004a). Role of histone H2A ubiquitination in Polycomb silencing. Nature *431*, 873–878.

Wang, L., Brown, J.L., Cao, R., Zhang, Y., Kassis, J.A., and Jones, R.S. (2004b). Hierarchical recruitment of polycomb group silencing complexes. Mol. Cell *14*, 637–646.

Wang, R., Taylor, A.B., Leal, B.Z., Chadwell, L.V., Ilangovan, U., Robinson, A.K., Schirf, V., Hart, P.J., Lafer, E.M., Demeler, B., et al. (2010). Polycomb group targeting through different binding partners of RING1B C-terminal domain. Structure *18*, 966–975.

Wu, X., Johansen, J.V., and Helin, K. (2013). Fbxl10/Kdm2b recruits polycomb repressive complex 1 to CpG islands and regulates H2A ubiquitylation. Mol. Cell *49*, 1134–1146.

Yuan, W., Wu, T., Fu, H., Dai, C., Wu, H., Liu, N., Li, X., Xu, M., Zhang, Z., Niu, T., et al. (2012). Dense chromatin activates Polycomb repressive complex 2 to regulate H3 lysine 27 methylation. Science *337*, 971–975.

Zhang, J., Benavente, C.A., McEvoy, J., Flores-Otero, J., Ding, L., Chen, X., Ulyanov, A., Wu, G., Wilson, M., Wang, J., et al. (2012). A novel retinoblastoma therapy from genomic and epigenetic analyses. Nature *481*, 329–334.

EXTENDED EXPERIMENTAL PROCEDURES

Generation of the De Novo Targeting System

A TetO array comprising 14 TetO sites interspersed by random CpG-free 21 bp sequences was synthesized by Invitrogen Gene Art. Using the pRED-ET/RPSL-Neo counter selection system (Gene Bridges) the TetO array was seamlessly recombineered into BAC RP11 419E6, which contains approximately 170 kb of DNA corresponding to a gene desert on human chromosome 7. Tol2 sequence elements (Shakes et al., 2011) were then recombineered into the plasmid backbone portion of the BAC, together with a mammalian neomycin resistance cassette. Using Lipofectamine 2000 (Life Technologies) the TetO BAC was cotransfected with a Tol2 transposase expression plasmid into E14 mouse ESCs, and stable G418 (400 μg/ml) resistant clones were obtained. A PCR screen was used to identify a clone exhibiting Tol2-mediated single-copy integration, and splinkerette PCR was used to map the precise BAC integration site on mouse chromosome 8.

The coding sequence for the TetR DNA-binding domain was PCR-amplified from pTet-tTS (Clontech) with the upstream primer engineered to contain 3x SV40 NLS. The 3xNLS-TetR fragment was inserted into a modified pCAG-IRES-Puro mammalian expression plasmid, between the coding sequence for an N-terminal FLAG STREPx2 (FS2) tag and a 3′ ligation-independent cloning (LIC) site. The resultant plasmid was named pCAGFS2TetR. Coding sequences for proteins of interest were PCR-amplified with primers engineered to contain appropriate LIC sequences, allowing LIC cloning into pCAGFS2TetR. The resultant plasmids expressed a fusion protein comprising FS2-TetR-protein of interest. All plasmids were transfected into the TetO BAC-containing ESCs using Lipofectamine 2000 (Life Technologies) and stable clones expressing the TetR fusion proteins were obtained following selection with puromycin (1 μg/ml) and G418 (400 μg/ml). TetR fusion proteins were detected in ChIP experiments using an FS2-specific antibody.

The RING1B PCGF4 catalytic domain fusion (RPCD) comprises amino acids 1-116 of RING1B and 3-109 of PCGF4 separated by a flexible 14 amino acid linker. The RPCD catalytic mutant includes point mutations at the E2 interface of RING1B (I53A), and within the PCGF4 RING finger (C51G).

Chromatin Immunoprecipitation

Chromatin immunoprecipitation was performed as described previously (Farcas et al., 2012), with minor modifications. Briefly, for nonhistone ChIP cells were fixed for 1 hr in 2 mM EGS, followed by 15 min in 1% formaldehyde, while for histone modification ChIP cells were fixed for 10 min in 1% formaldehyde alone. In both cases, formaldehyde was quenched by the addition of glycine to a final concentration of 125 μM.

Sonication was performed using a BioRuptor sonicator (Diagenode, Liege, Belgium) to produce fragments of approximately 0.5–1 kb. Immunoprecipitation was performed overnight at 4°C with approximately 3 μg of antibody and chromatin corresponding to 5×10^6 cells (nonhistone and H2AK119ub1 ChIPs) or 1×10^6 cells (histone ChIPs other than H2AK119ub1). Antibody-bound chromatin was isolated on protein A agarose beads (RepliGen, Waltham, CA) or protein A magnetic Dynabeads (Invitrogen, Carlsbad, CA) and washes were performed with low-salt buffer (0.1% SDS, 1% Triton, 2 mM EDTA, 20 mM Tris-HCl (pH 8.1), 150 mM NaCl), high-salt buffer (0.1% SDS, 1% Triton, 2 mM EDTA, 20 mM Tris-HCl (pH 8.1), 500 mM NaCl), LiCl buffer (0.25 M LiCl, 1% NP40, 1% Deoxycholate, 1 mM EDTA, 10 mM Tris-HCl [pH 8.1]) and TE buffer (x2) (10 mM Tris-HCl (pH 8.0), 1 mM EDTA). To prepare ChIP-seq material, ChIP DNA was eluted, and crosslinks reversed at 65°C, then samples were then sequentially treated with RNase and proteinase K before being purified on a PureLink PCR micro column (Invitrogen). Alternatively, to prepare ChIP material for qPCR, following ChIP washes beads and antibody-bound chromatin were boiled for 10 min in 10% Chelex 100 Resin (BioRad) and treated with proteinase K for 30 min at 55°C. Samples were then boiled for a further 10 min to inactivate proteinase K. Beads were collected by centrifugation and supernatant was retained for qPCR analysis. ChIP qPCR data were expressed as either a percentage of input (y axis labeled "% Input") or enrichment relative to a control polycomb target site (y axis labeled "rel. enrichment").

ChIP-sequencing libraries were generated as described previously (Blackledge et al., 2010) and sequenced on the Illumina HiSeq2000 platform with 51 bp reads. For *Ring1a*$^{-/-}$*Ring1b1b*$^{fl/fl}$ cells, ChIP-sequencing experiments for RING1B, SUZ12, EZH2 and K27me3 were performed in biological duplicate, while for *Kdm2b*$^{fl/fl}$ cells KDM2B and RING1B ChIP-seq was done in biological triplicate and SUZ12 in biological duplicates.

Antibodies used for ChIP were as follows:

Antigen	Source of Antibody
KDM2B	Described previously (Farcas et al., 2012)
RING1B	Described previously (Atsuta et al., 2001)
EZH2	Cell Signaling 5246S
SUZ12	Cell Signaling 3737S
PCGF1	Generated for the current study
PCGF2	Santa Cruz sc-10744x
CBX7	Abcam ab21873

(*Continued on next page*)

Continued

Antigen	Source of Antibody
FLAG STREPX2	Described previously (Farcas et al., 2012)
H3K27me3	Diagenode C15410069 (pAb-069)
H2AK119ub1	Described previously (Farcas et al., 2012)
HisH3	Described previously (Farcas et al., 2012)
H3K9ac	Millipore 07-352
H4K20me3	Abcam ab9053
H3K9me3	Abcam ab8898
H3K4me3	Described previously (Farcas et al., 2012)
RNA Pol II	Covance 8WG16

PCGF1 Antibody

A polyclonal antibody against PCGF1 (sequence MASPQGGQIAIAMRLRNQLQSVYKMDPLRNEEEVR) was generated by immunization of a rabbit with a synthetically synthesized peptide conjugated to mariculture KLH carrier protein (ThermoScientific, Waltham, MA). PCGF1 peptide was covalently immobilized on SulfoLink resin (ThermoScientific) and antibody was affinity purified prior to use.

PCGF1 Knockdown

Control or PCGF1 shRNAs were cloned into pLKO.1blasticidin (Addgene, Cambridge, MA) and sequence verified. To produce recombinant lentiviral particles, the shRNA constructs were cotransfected with psPAX2 packaging plasmid and pMD2.G envelope helper plasmid into 293T cells using FuGene (Roche, Basel, Switzerland). The 21-mer shRNA sequences used were: (PCGF1) 5′-CCCAGATCACATGACAATGAA −3′, (control) 5′-CCTAAGGTTAAGTCGCCCTCG-3′. Lentiviral infection of cells was performed overnight in the presence of 4 μg/ml polybrene. The following day, cells were diluted into fresh growth media and allowed to settle onto gelatine-coated dishes. Blasticidin selection (10 μg/ml) was started 48 hr after transduction and stable lines were isolated, expanded and screened for stable PCGF1 knockdown.

Gene Expression Analysis

Total RNA was extracted using the QIAGEN RNeasy Mini kit. Approximately 10 μg RNA was treated with Turbo DNase (Ambion, Carlsbad, CA) at 37°C for 30 min, according to the manufacturer's instructions. Genomic DNA-free RNA samples were further purified using the RNeasy kit RNA cleanup protocol. Samples were run on a 1% agarose gel to check quality of RNA preparation and integrity of 18S and 28S rRNA bands. For RT-PCR analysis, cDNA was synthesized with the ImProm-II Reverse Transcription System (Promega, Madison, WI). Quantitative real-time PCR was performed with Quantace SYBR Green master mix, using *Gapdh* or *Hprt1* housekeeping genes for normalization. For mRNA-seq analysis 1 μg of purified total RNA was used to first isolate polyA plus RNA and then a directional library was prepared using the NEBNext UltraTM Directional RNA library Prep Kit for Illumina (NEB, Ipswich, MA).

Protein Complex Purification Followed by Liquid Chromatography and Tandem Mass Spectrometry Analysis

Tandem mass spectrometry (LC–MS/MS) and data analyses were performed as described previously (Farcas et al., 2012). Briefly, to identify KDM2B short form (SF) associated proteins, a feeder-independent mouse ESC lines expressing Flag/2xStrepII-tagged KDM2B SF was generated. To verify whether KDM2BΔCxxC retains the capacity to associate with PCGF1/PRC1 variant complex, E14 ESC lines expressing Flag/2xStrepII tagged wild-type (WT) KDM2B and KDM2BΔCxxC were produced. 15 mg of nuclear extract (corresponding to approximately 100 confluent 15 cm dishes) was used for each affinity purification. Affinity purification from cells expressing full-length TetR-PCGF4, the TetR-PCGF4/RING1B minimal catalytic fusion or TetR alone was carried out using the same purification approach. Following elution with desthiobiotin, the fractions collected were precipitated using chloroform/methanol and the protein pellets resuspended and subjected to in-solution tryptic digestion followed by nano-liquid chromatography-tandem mass spectrometry (nLC-MS/MS) analysis using a nano-Acquity UPLC (Waters) coupled to an Orbitrap Velos/Elite mass spectrometer (Thermo). MS/MS spectra were searched against the UniProt SwissProt Mouse database (16,683 sequences) in Mascot v2.3.01, allowing one missed cleavage and 20 ppm/0.5 Da mass deviations in MS/MSMS, respectively. Carbamidomethylation of cysteine was a fixed modification. Oxidation of methionine, and deamidation of asparagine and glutamine residues were used as variable modifications. Protein assignment was based on at least two peptides identified. Mascot scores and peptide coverage are indicated for each protein hit.

ChIP-Seq and RNA-Seq Analysis

ChIP-seq reads for the antibody IPs or an input DNA sample were aligned to the mouse mm9 genome release using bowtie v1.1.2. The default alignment parameters were used in all cases with the exception that reads which could be aligned to more than one site in the genome were supressed (-m 1). When comparing ChIP-seq for untreated and tamoxifen-treated cell lines (*Ring1a*$^{-/-}$*Ring1b*$^{fl/fl}$

and $Kdm2b^{fl/fl}$) each replicate was down-sampled to the number of reads contained in the biological replicate with the lowest total number of aligned reads.

To identify high-confidence RING1B-bound intervals, we compared the $Ring1a^{-/-}Ring1b^{fl/fl}$ cells before and after tamoxifen treatment to identify sites which showed significant loss of RING1B ChIP-seq signal. This was achieved using duplicate experiments comparing untreated and tamoxifen-treated $Ring1a^{-/-}Ring1b^{fl/fl}$ ChIP-seq in diffReps (v1.55.1) with paired input controls. To identify SUZ12 and EZH2 interval sets, the independent ChIP-seq replicates from the untreated $Ring1a^{-/-}Ring1b^{fl/fl}$ cells were submitted to MACS (v1.4) with matched input controls. Interval sets for biological replicates were combined using BedTools (Quinlan and Hall, 2010) and only intervals identified in both replicates were considered for further analysis.

RING1B, SUZ12 and EZH2 interval sets were annotated with normalized read density counts for untreated and tamoxifen-treated samples from both $Ring1a^{-/-}Ring1b^{fl/fl}$ and $Kdm2b^{fl/fl}$ experiments, and the untreated to treated fold change was calculated. Interval sets were visualized and filtered using Multi-Image Genome viewer (McGowan et al., 2013). Heat maps were generated using HOMER (Heinz et al., 2010) and visualized with Java TreeView (Saldanha, 2004). To consider changes in polycomb protein occupancy at bivalent gene promoters, we obtained previously published bivalent gene annotation for a set of 15,404 genes (Brookes et al., 2012; Mikkelsen et al., 2007). Transcription start sites (TSS) ± 500 bp were annotated with normalized read density counts and fold changes from ChIP-seq experiments in the $Ring1a^{-/-}Ring1b^{fl/fl}$ cells. REST peaks (GSE27148) were identified using MACS as described for SUZ12 and EZH2 above (Arnold et al., 2013) and were intersected with SUZ12 peaks using BedTools.

For RNA-seq analysis, paired end 51-bp reads were aligned to the mm9 reference genome using Tophat2 (v0.5) with default parameters. RPKM values were obtained from biological triplicates of the $Kdm2b^{fl/fl}$ and 72 hr tamoxifen treated cells using cuffdiff (v2.1.1) on an mm9 refGene set obtained from the UCSC table browser. For RNA-seq analysis each RING1B interval was annotated with the closest gene, and the fold change between mean RPKM values for treated and untreated $Kdm2b^{fl/fl}$ cells was determined.

Statistical Analysis

Fold changes in ChIP-seq and gene expression changes were tested for significance with the Welch Two Sample T-Test using R (version 3.0.1). Correlations were tested using Pearson's Product-Moment Correlation statistic and linear regression analyses were performed using a standard linear regression algorithm in R.

Kdm2b Knockout Mouse and ESC Generation

MS12 ESCs were used for producing a conditional knockout allele for *Kdm2b*. The arms of homology span a HindIII site at mm9 chr5; 123,330,124 to an EcoRI site at 123,340,540. A Neomycin resistant gene flanked with FRT and loxP sequences was inserted at KpnI site (123,338,886) and a loxP sequence at SalI site (123,338,409). ESCs were treated with flippase expression plasmid to remove the Neomycin resistant gene, and then injected into mouse blastocysts to establish $Kdm2b^{fl/fl}$ mouse. ES cell lines harboring the allele $Kdm2b^{fl/fl}$*:Gt(Rosa26)Sor-ERT2Cre+/−* were raised from the mating between $Kdm2b^{wt/fl}$*:Gt(Rosa26)Sor-ERT2Cre+/−* and $Kdm2b^{fl/fl}$.

Bisulfite Sequencing

Bisulfite sequencing was performed using the EZ DNA Methylation-Gold Kit (Zymo Research) following manufacturer's instructions. PCR-amplified DNA was cloned into pGEM-T Easy (Promega) and sequenced.

SUPPLEMENTAL REFERENCES

Atsuta, T., Fujimura, S., Moriya, H., Vidal, M., Akasaka, T., and Koseki, H. (2001). Production of monoclonal antibodies against mammalian Ring1B proteins. Hybridoma *20*, 43–46.

Heinz, S., Benner, C., Spann, N., Bertolino, E., Lin, Y.C., Laslo, P., Cheng, J.X., Murre, C., Singh, H., and Glass, C.K. (2010). Simple combinations of lineage-determining transcription factors prime cis-regulatory elements required for macrophage and B cell identities. Mol. Cell *38*, 576–589.

McGowan, S.J., Hughes, J.R., Han, Z.P., and Taylor, S. (2013). MIG: Multi-Image Genome viewer. Bioinformatics *29*, 2477–2478.

Mikkelsen, T.S., Ku, M., Jaffe, D.B., Issac, B., Lieberman, E., Giannoukos, G., Alvarez, P., Brockman, W., Kim, T.K., Koche, R.P., et al. (2007). Genome-wide maps of chromatin state in pluripotent and lineage-committed cells. Nature *448*, 553–560.

Quinlan, A.R., and Hall, I.M. (2010). BEDTools: a flexible suite of utilities for comparing genomic features. Bioinformatics *26*, 841–842.

Saldanha, A.J. (2004). Java Treeview—extensible visualization of microarray data. Bioinformatics *20*, 3246–3248.

Shakes, L.A., Abe, G., Eltayeb, M.A., Wolf, H.M., Kawakami, K., and Chatterjee, P.K. (2011). Generating libraries of iTol2-end insertions at BAC ends using loxP and lox511 Tn10 transposons. BMC Genomics *12*, 351.

Cell

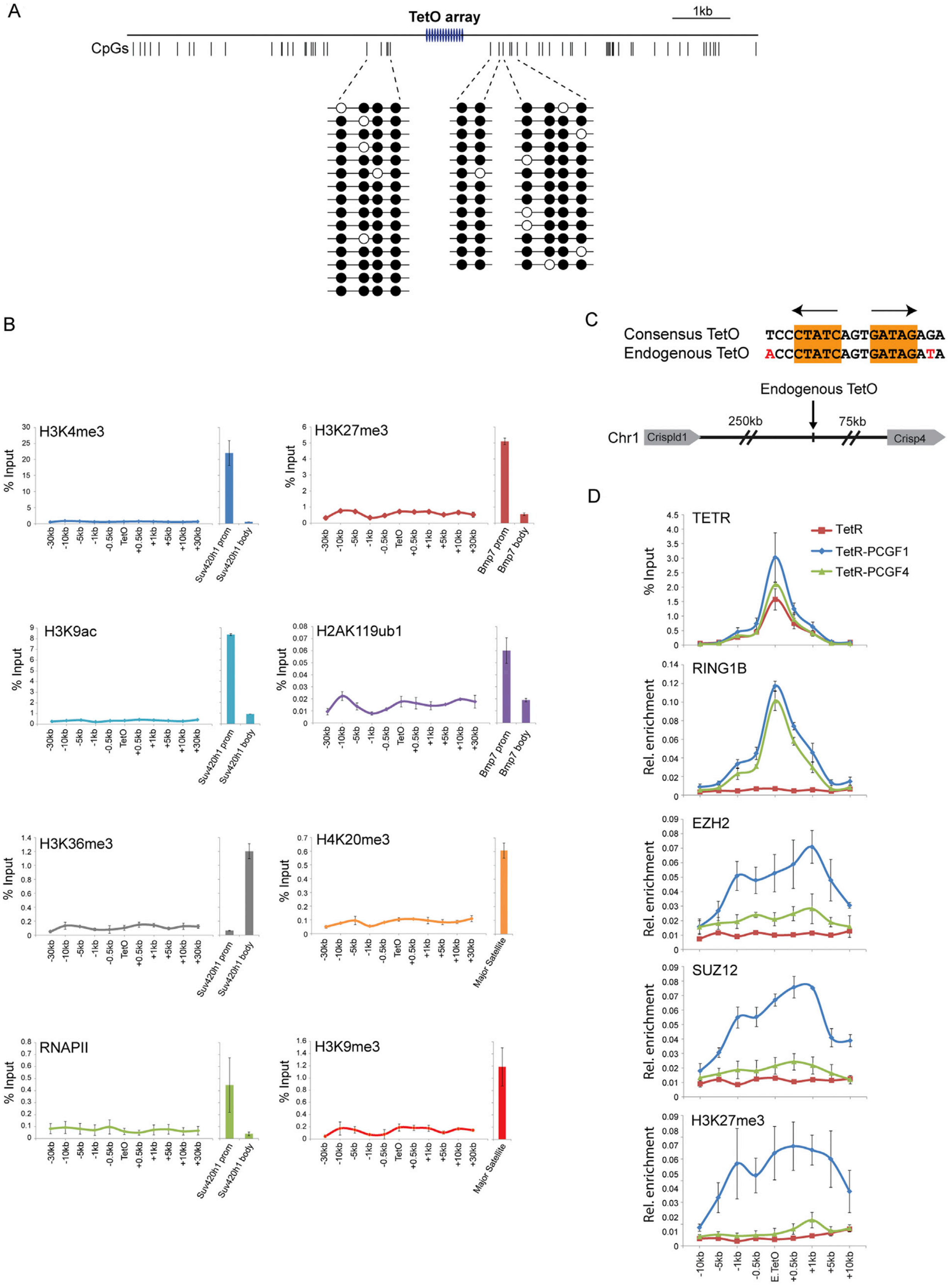

(legend on next page)

Figure S1. Variant PRC1 Complex-Dependent PRC2 Targeting Occurs on an Inert TetO Array Template and at a Single Natural TetO Site in Mouse ESCs, Related to Figure 1

(A) Bisulfite sequencing reveals that CpG dinucleotides flanking the engineered TetO array in the Human BAC DNA remain methylated, as they are in human tissue, when stably inserted into the mouse genome. This indicates that normal DNA methylation features are recapitulated on the integrated DNA sequence.

(B) ChIP-qPCR analysis of the human BAC/TetO array stably integrated into mouse ESCs. ChIP was performed with antibodies specific for permissive chromatin marks and features of active transcription (left: H3K4me3, H3K9ac, H3K36me3, and RNAPII) and repressive chromatin features (right: H3K27me3, H2AK119ub1, H4K20me3, and H3K9me3). For each antibody, qPCR enrichment at a known target site is shown as a positive control. For gene-associated chromatin modifications this includes promoter (prom) and body amplicons for the indicated genes, while H3K9me3 and H4K20me3 were analyzed at sites in the repetitive major satellite regions. Together these observations indicate that the human BAC DNA, when inserted into mouse ESCs, retains its inert features and remains suitable for studying polycomb domain formation in tethering assays.

(C) A position on mouse chromosome 1, distant from surrounding genes, contains a single site that has a high degree of homology to a consensus bacterial TetO-binding site. This provides a "natural" site in the genome at which to analyze TetR fusion protein binding and function.

(D) ChIP-qPCR analysis of TetR, TetR-PCGF1 (variant PRC1), and TetR-PCGF4 (canonical PRC1) binding at the single natural TetO and flanking regions (Top). In each case, occupancy of PRC1 leads to RING1B recruitment (second panel). However, as observed at the engineered TetO array EZH2, SUZ12, and H3K27me3 are only recruited to the endogenous TetO site when a variant PRC1 complex occupies the site (bottom three panels). Together these observations demonstrate that variant PRC1 complex-dependent PRC2 recruitment and de novo polycomb domain formation is observed at a "natural" sequence in the mouse genome, and is not the result of copy number or site specific features inherent to the engineered TetO array. All ChIP experiments in (B and D) were performed in biological duplicate with error bars showing SEM.

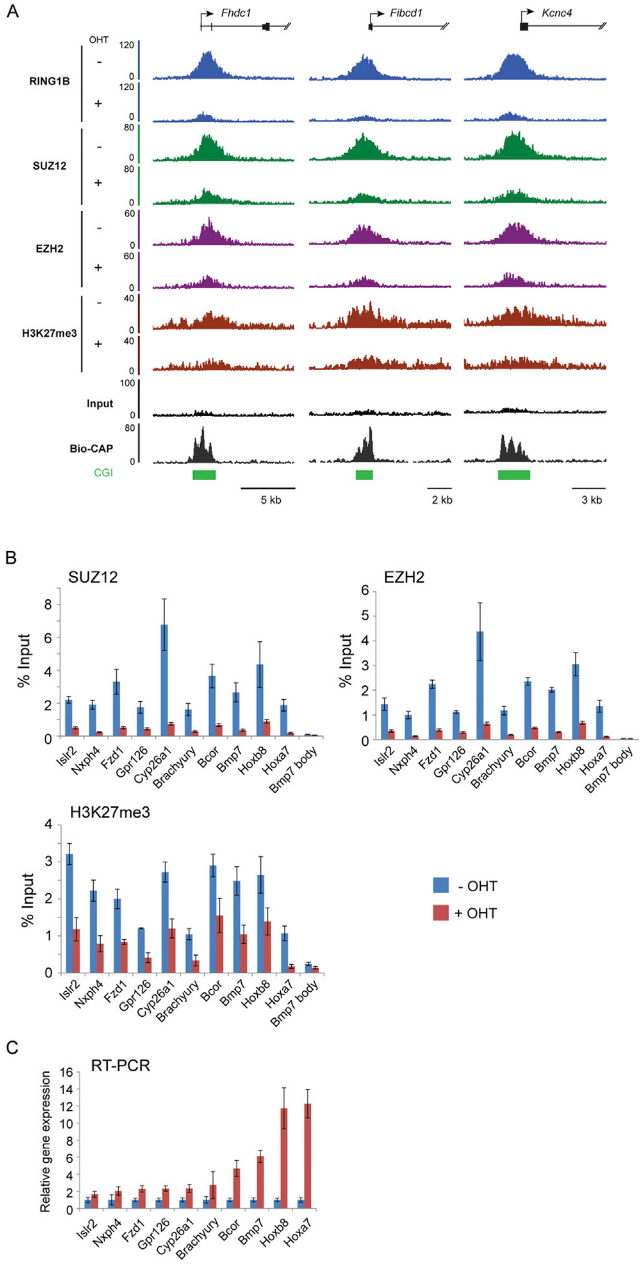

Figure S2. Conditional Deletion of RING1A/B Results in Depletion of H2AK119ub1, Loss of PRC2 Occupancy, and H3K27me3 at Target Genes Independently of the Magnitude of Associated Gene Expression Change, Related to Figure 4

(A) Snapshots of ChIP-seq traces for RING1B, SUZ12, EZH2 and H3K27me3 in the *Ring1a*$^{-/-}$*Ring1b*$^{fl/fl}$ cells prior to (−OHT) and following 48 hr (+OHT) of tamoxifen treatment. Three representative genes are depicted illustrating the reduction in RING1B, SUZ12, EZH2, and H3K27me3 following removal of the RING1A/B. A Bio-CAP sequencing trace is shown to indicate the location of nonmethylated DNA and a CpG Island (CGI) prediction annotation track is show as green bars under the traces. This complements examples already shown in main Figure 4.

(B) ChIP-qPCR analysis for PRC2 components SUZ12, EZH2, and H3K27me3 at a series of sites showing loss of these factors in the ChIP-seq analysis in main Figure 4 after tamoxifen treatment. All ChIP experiments in (C-D) were performed in biological triplicate with error bars showing SEM.

(C) Gene expression analysis for the polycomb target genes analyzed by ChIP in (B). There is no correlation between gene expression change and scale of polycomb group protein loss, suggesting that altered gene expression is not driving PRC2 loss. RT-PCR was performed in biological triplicate and is normalized to *Hprt1* expression. Error bars show SEM.

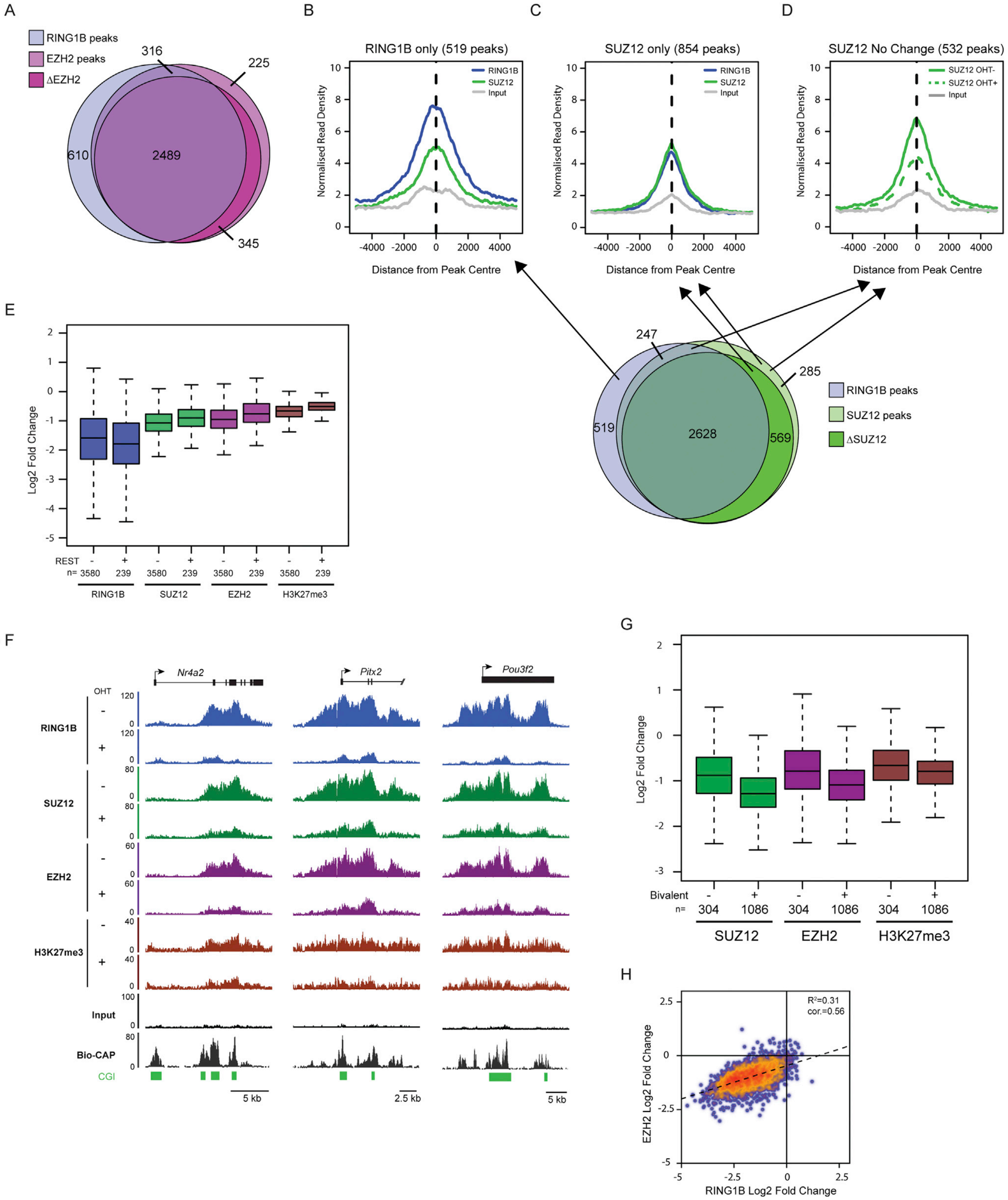

(legend on next page)

Figure S3. PRC1 Has a Genome-wide-Role in PRC2 Recruitment and Polycomb Domain Formation at Target Sites in Mouse ESCs, Related to Figure 4

(A) A Venn diagram showing the overlap of RING1B (light blue) and EZH2 (light pink) peaks including a further segregation of EZH2-bound regions that show a greater than 1.5-fold change in EZH2 occupancy (ΔEZH2, dark pink) after RING1A/B deletion. The majority of EZH2-bound locations show changes in EZH2 signal following removal of RING1A/B, and most of the changes are restricted to sites associated with RING1B peaks.

(B) A metaplot of RING1B and SUZ12 ChIP-seq data at RING1B peaks that do not overlap with SUZ12 sites identified by peak calling. This indicates that these regions exhibit SUZ12 enrichment but are likely below the enrichment level required for peak detection.

(C) A metaplot of RING1B and SUZ12 ChIP-seq data at SUZ12 peaks that do not overlap with RING1B sites identified by peak calling. This indicates that these regions exhibit RING1B enrichment but are likely below the enrichment level required for peak detection.

(D) A metaplot of SUZ12 ChIP-seq data in the *Ring1a*$^{-/-}$*Ring1b*$^{fl/fl}$*b* cells at SUZ12 peaks exhibiting a less than 1.5-fold reduction in SUZ12 signal following tamoxifen treatment. Importantly these sites still show reduction in SUZ12 signal suggesting that loss of RING1A/B affects PRC2 occupancy at most sites.

(E) A box and whisker plot indicating the Log2 fold change in polycomb factors at SUZ12-bound sites with and without REST following loss of RING1A/B. This indicates that loss of PRC2 occurs at REST-bound sites in the absence of PRC1.

(F) Snapshots of ChIP-seq traces for RING1B, SUZ12, EZH2 and H3K27me3 in the *Ring1a*$^{-/-}$*Ring1b*$^{fl/fl}$ cells prior to (−OHT) and following 48 hr (+OHT) of tamoxifen treatment at sites previously reported to rely on the Meg3 long noncoding RNA for PRC2 targeting. In all cases we observe appreciable loss of PRC2 following RING1A/B deletion indicating that Meg3-dependent targeting is not sufficient to maintain normal levels of PRC2 at these sites.

(G) A box and whisker plot indicating the Log2 fold change in PRC2 factors and H3K27me3 at sites considered to be bivalent. Bivalent sites appear to have slightly larger fold changes in PRC2 occupancy following RING1A/B deletion.

(H) A scatter plot comparing the fold change of RING1B and EZH2 at EZH2 peaks. This indicates a clear correlation between the magnitude in RING1B and EZH2 change suggesting these changes may be mechanistically linked.

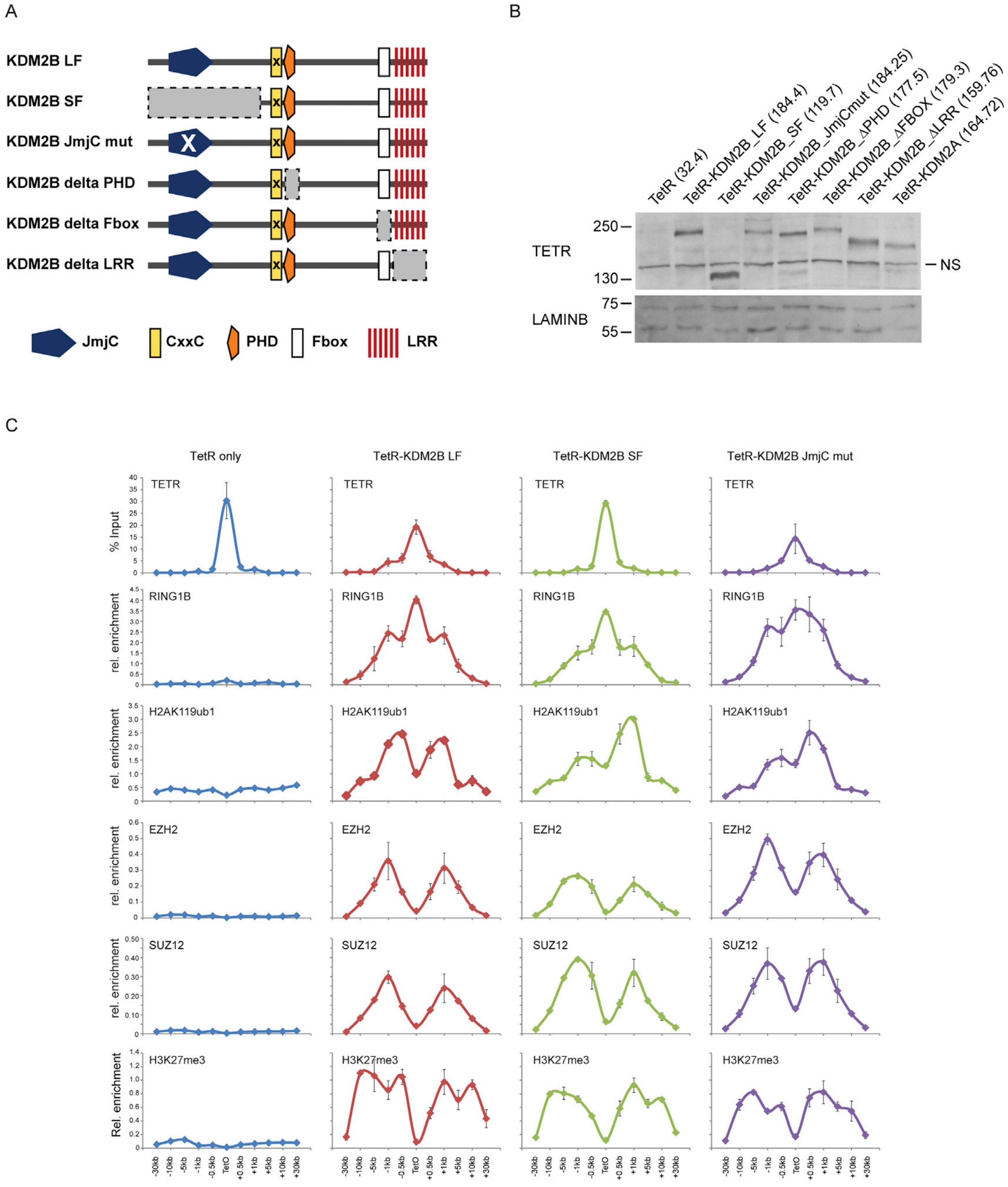

D

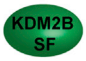

	KDM2B SF IP	
Name	Score	Cov (%)
KDM2B SF	4255	25
RING1B	279	31
BCORL1	393	8
BCOR	273	13
PCGF1	102	38
RYBP	86	14
YAF2	17	13
SKP1	1677	74

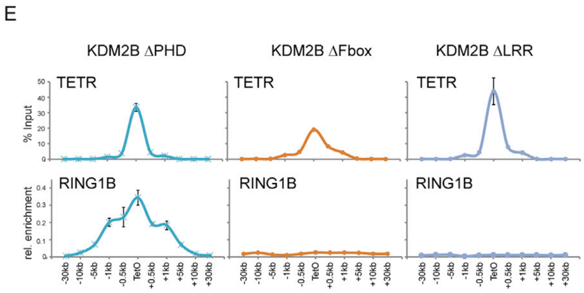

(legend on next page)

Figure S4. Both the Short and Long Form of KDM2B Mediate Polycomb Domain Formation in a Histone Demethylase Activity-Independent Manner, Related to Figure 5

(A) KDM2B long form (LF) and short form (SF) with their domain organization indicated. Additional, TetR fusion constructs which have had domains removed (gray boxes) or mutated are indicated.

(B) Western blot analysis of the TetR-KDM2B fusion cell lines indicating roughly equal protein expression.

(C) ChIP-qPCR analysis for the TetR fusion protein, RING1B, H2AK119ub1, SUZ12, EZH2, and H3K27me3 across the TetO containing region in the TetR only, TetR-KDM2B LF, TetR-KDM2B SF, and TetR-KDM2B LF demethylase mutant (JmjC mutant). All three versions of KDM2B lead to efficient RING1B recruitment, H2AK119ub1, and formation of a polycomb domain containing SUZ12, EZH2, and H3K27me3. This indicates that both forms of KDM2B can form polycomb domains independent of their demethylase activity.

(D) An epitope tagged version of the KDM2B-SF was stably expressed in mouse ESCs, affinity purified, and associated proteins identified by tandem mass spectrometry. This revealed that the short form of KDM2B forms the same variant PRC1 complex as the long form of the protein, consistent with its capacity to recruit RING1B and form polycomb domains in tethering assays.

(E) Based on the capacity of KDM2B-SF to associate with the PCGF1/PRC1 complex (D) the domain(s) mediating this were further mapped in tethering assays. The C-terminal Fbox and LRR domains are required for RING1B recruitment whereas the PHD domain is dispensable.

A B

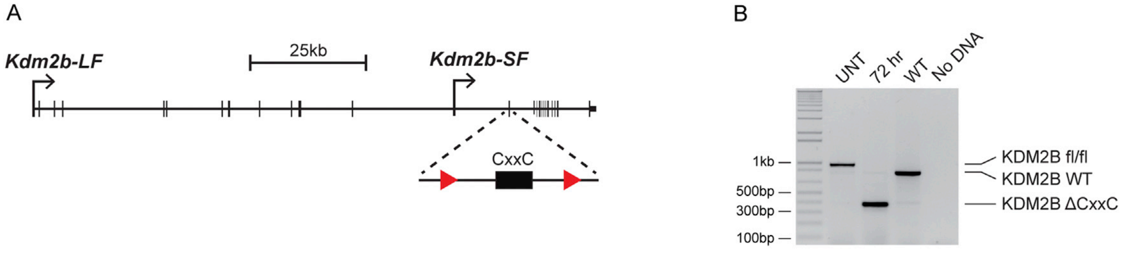

C

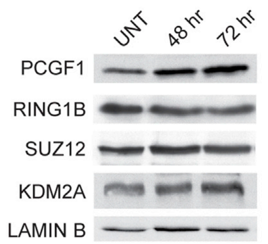

D

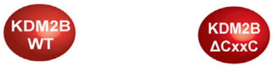

	KDM2B WT		KDM2B ΔCxxC	
Name	Score	Cov (%)	Score	Cov (%)
KDM2B	36406	64	28907	66.3
SKP1	3188	73.6	8034	87.7
BCOR	413	12.2	5177	62.4
BCORL1	124	5.9	872	21.4
RING1B	259	33.9	1561	68.8
PCGF1	113	32.8	596	58.7
RYBP/YAF2	87	9.6	202	25.9

Figure S5. A Model Cell System to Inducibly Disrupt Targeting of the PCGF1/PRC1 Complex, Related to Figure 5

(A) A schematic of the *Kdm2b* gene showing the long form (*Kdm2b-LF*) and short form (*Kdm2b-SF*) transcription start sites. The positions of LoxP sites are highlighted flanking the exon which encodes the ZF-CxxC domain.

(B) PCR with primers spanning the floxed exon was performed on genomic DNA from the *Kdm2b*$^{fl/fl}$ cells (UNT), the *Kdm2b*$^{fl/fl}$ cells treated for 72 hr with tamoxifen (72 hr), and wild-type cells (WT). 72hrs of tamoxifen treatment leads to a clear deletion of the floxed exon.

(C) Western blot analysis indicates that loss of the KDM2B ZF-CxxC domain does not lead to destabilization of the PCGF1 and RING1B components of the KDM2B variant PRC1 complex or upregulation of its paralogue KDM2A. Furthermore, PRC2 remains present as indicated by normal levels of SUZ12.

(D) Affinity purification of full-length epitope tagged wild-type (WT) KDM2B and KDM2BΔCxxC followed by tandem mass spectrometry-based analysis of associated proteins. The mascot score and percentage coverage is indicated for the KDM2B/PRC1 complex components. Importantly, removal of the ZF-CxxC exon generates a product that still associates with the PCGF1/PRC1 variant complex but lacks its capacity to bind nonmethylated DNA.
